# Thermoelectric Detection of Crossed Andreev Reflections in Quantum Hall/Superconductor Hybrid Structures

**DOI:** 10.1038/s41467-026-74064-2

**Published:** 2026-06-05

**Authors:** Jiashu Wang, Sina Ahadi, Boliang Liu, Leon Balents, Simon Munyan, Susanne Stemmer

**Affiliations:** 1 Materials Department, University of California, Santa Barbara, CA 93106-5050 USA; 2 Kavli Institute for Theoretical Physics, University of California, Santa Barbara, CA 93106-4030 USA

**Keywords:** Quantum Hall, Superconducting properties and materials

## Abstract

Hybrid structures that couple the chiral edge states of a quantum Hall insulator to an *s*-wave superconductor have emerged as a promising platform for non-Abelian quasiparticles and Cooper pair splitters. These devices can feature crossed Andreev reflections (CAR) that convert electrons from chiral edge modes that are upstream from the superconductor into holes that are injected into the downstream modes. Here, we interface the quantum Hall edge modes of cadmium arsenide (Cd_3_As_2_) films with a thin superconducting NbN strip. We observe oscillations in magnetic fields in the downstream resistance and thermoelectric response. While electron-like carriers dominate the sign of the ohmic response, likely due to the large elastic co-tunneling contribution, the downstream thermoelectric response alternates in sign. The results point to thermoelectric measurements as a promising probe to elucidate the competing nature of Andreev and tunneling processes at quantum Hall/superconductor interfaces.

## Abstract (web summary)

The authors interface the quantum Hall edge modes of Cd_3_As_2_ films with a thin superconducting NbN strip. They find that the downstream thermoelectric response alternates in sign, revealing the contribution of crossed Andreev reflections.

## Introduction

Topological superconductors that host non-Abelian quasiparticles have attracted considerable attention due to their potential for applications in fault-tolerant quantum computing^1–3^. A promising platform involves coupling of the chiral edge states of a quantum Hall insulator to an *s*-wave superconductor^4–7^. Experimental studies of induced superconductivity in this platform have focused on device geometries that allow for chiral Andreev edge states (CAES)^8–15^ or crossed Andreev reflections (CAR)^16–18^.

**Fig. 1:**
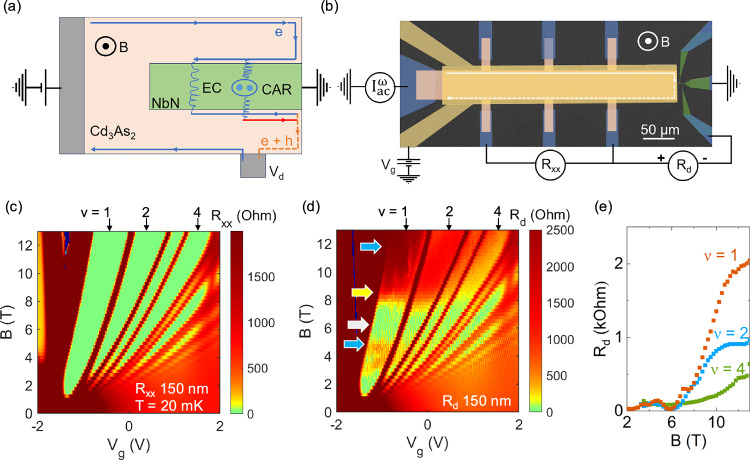
Measurement configuration and results from a device with a 150 nm wide NbN finger. **a** Schematic of elastic co-tunneling (ECT) and crossed Andreev reflection (CAR) processes in a quantum Hall/superconductor structure, after ref. ^22^. **b** False-color scanning electron microscope image of the device. A narrow NbN finger (green) interfaces the edge states of a Cd₃As₂ Hall bar (pink), while a top gate (yellow) tunes the chemical potential. Chiral edge states propagate clockwise for positive fields. The downstream resistance *R*_d_ is measured between a normal contact and the NbN drain. **c**, **d** Landau level maps of the longitudinal resistance *R*_*xx*_ and downstream resistance *R*_d_, as a function of magnetic field *B* and gate voltage *V*_*g*_, measured on a device with a 150 nm wide NbN finger. Arrows in (d) mark regimes of different *R*_d_, for comparison with Fig. 3(b). **e** *R*_d_ values at the center of different Hall plateaus as a function of magnetic field *B*. The *v* = 3 plateau is not included due to insufficient quantization.

A prototype device for inducing CAR into a quantum Hall insulator involves a thin superconducting strip that separates the left and right moving chiral edge modes (Fig. 1a)^16^. In a standard Hall bar geometry, with the narrow superconducting finger acting as the drain, CAR can be detected as a negative downstream resistance (*R*_d_) in the measurement configuration shown in Fig. 1b. A negative downstream resistance indicates that electron-like quasiparticles from the chiral edge states form Cooper pairs in the superconductor, resulting in the injection of holes into the chiral edge modes that are downstream from the superconductor. The probability of CAR decays with increasing width of the superconducting strip, which should not be much greater than the superconducting coherence length. Moreover, the superconducting strip must have a sufficiently large critical field to be compatible with the quantum Hall effect.

To date, negative values for *R*_d_, which would be consistent with CAR, have been reported for graphene quantum Hall systems^16,17^ and a quantum anomalous Hall insulator^18^. Theory, however, indicates that the probability of another process, elastic co-tunneling (ECT), is typically at least equal to that of CAR^19–22^. ECT does not involve Cooper pairing or splitting. It yields a positive downstream resistance, which obscures any negative CAR contribution. Although several theoretical studies have attempted to reconcile the larger ECT probability with observations of negative downstream resistances^22–25^, no consensus exists yet. To advance this important platform and to improve our understanding of the microscopic parameters that control CAR, additional materials systems and experimental probes would be highly desirable.

In this work, we investigate hybrid structures that employ cadmium arsenide (Cd_3_As_2_) films in the quantum Hall regime, whose edge states are interfaced with a thin superconducting NbN strip. Thin films of Cd_3_As_2_ are narrow-gap insulators that reach the ν = 1 quantum Hall state at fields below the critical field of NbN^26^. Spin-orbit coupling in these films is large^27^, which is a requirement for CAR^28^. We find that the downstream resistance oscillates as a function of the magnetic field. We use the heat-conducting properties of the chiral edge states to establish a temperature gradient across the superconductor and measure the downstream thermoelectric response. The thermoelectric voltage shows sign-alternating oscillations in a magnetic field. We suggest that a positive downstream thermoelectric voltage may indicate hole-like carriers in the downstream edge modes.

## Results and discussion

Figure 1b shows a fabricated device. Information on the superconducting properties of the NbN fingers is provided in Supplementary Note 1. In the absence of CAR or ECT, the upstream edge modes equilibrate at the drain, the downstream edge is on the same potential as the drain, and *R*_d_ equals the longitudinal resistance *R*_*xx*_. With CAR and/or ECT, Landauer-Büttiker theory predicts that *R*_d_ is modified according to^12,13,16,18,29^:

1 $${R}_{{{\rm{d}}}}=\frac{{P}_{{{\rm{e}}}}-{P}_{{{\rm{h}}}}}{1-{P}_{{{\rm{e}}}}+{P}_{{{\rm{h}}}}}\frac{h}{v{e}^{2}},$$

where *P*_e_ and *P*_h_ are the fractions of electrons and holes injected into the downstream edge modes, *h* is Planck’s constant, *e* is the electron charge, and *v* is the filling factor.

Figure 1c shows a Landau level map of *R*_*xx*_ for a device with a 150 nm wide NbN strip, obtained by sweeping *V*_g_ at different magnetic fields. At sufficiently large fields, peaks in *R*_*xx*_ appear when the Fermi level is in a Landau level, while *R*_*xx*_ vanishes for integer values of *v*, as expected for well-quantized Hall plateaus. At negative *V*_g_, past the *v* = 1 Hall plateau, the Fermi level enters the charge neutrality gap (for a detailed discussion, see ref. ^26^). Figure 1d shows the corresponding map for *R*_d_. Unlike *R*_*xx*_, *R*_d_ is not zero everywhere in the quantum Hall plateaus. As indicated by the arrows in Fig. 1d, regions of positive *R*_d_ alternate with regions where *R*_d_ ~ 0. In the *v* = 1 plateau, *R*_d_ ~ 0 at low fields. With increasing field, *R*_d_ shows a positive peak around 4 T, followed by a region of vanishing *R*_d_. At fields greater than 6 T *R*_d_ becomes positive again. The oscillatory behavior of *R*_d_ was reproducible in repeated measurements of the same device and observed in many different devices, provided that the NbN strips were narrower than 200 nm. Within the *v* = 1 plateau, oscillations are nearly independent of *V*_g_. The oscillations are weaker (or absent) in Hall plateaus with *v* > 1, while the large positive *R*_d_ at high fields is still observed. In addition, when *R*_d_ > 0, we observe a strong bias-dependence of *R*_d_ (Supplementary Note 2) that is similar to that found in normal metal-superconductor-normal metal systems^20,30,31^.

**Fig. 2:**
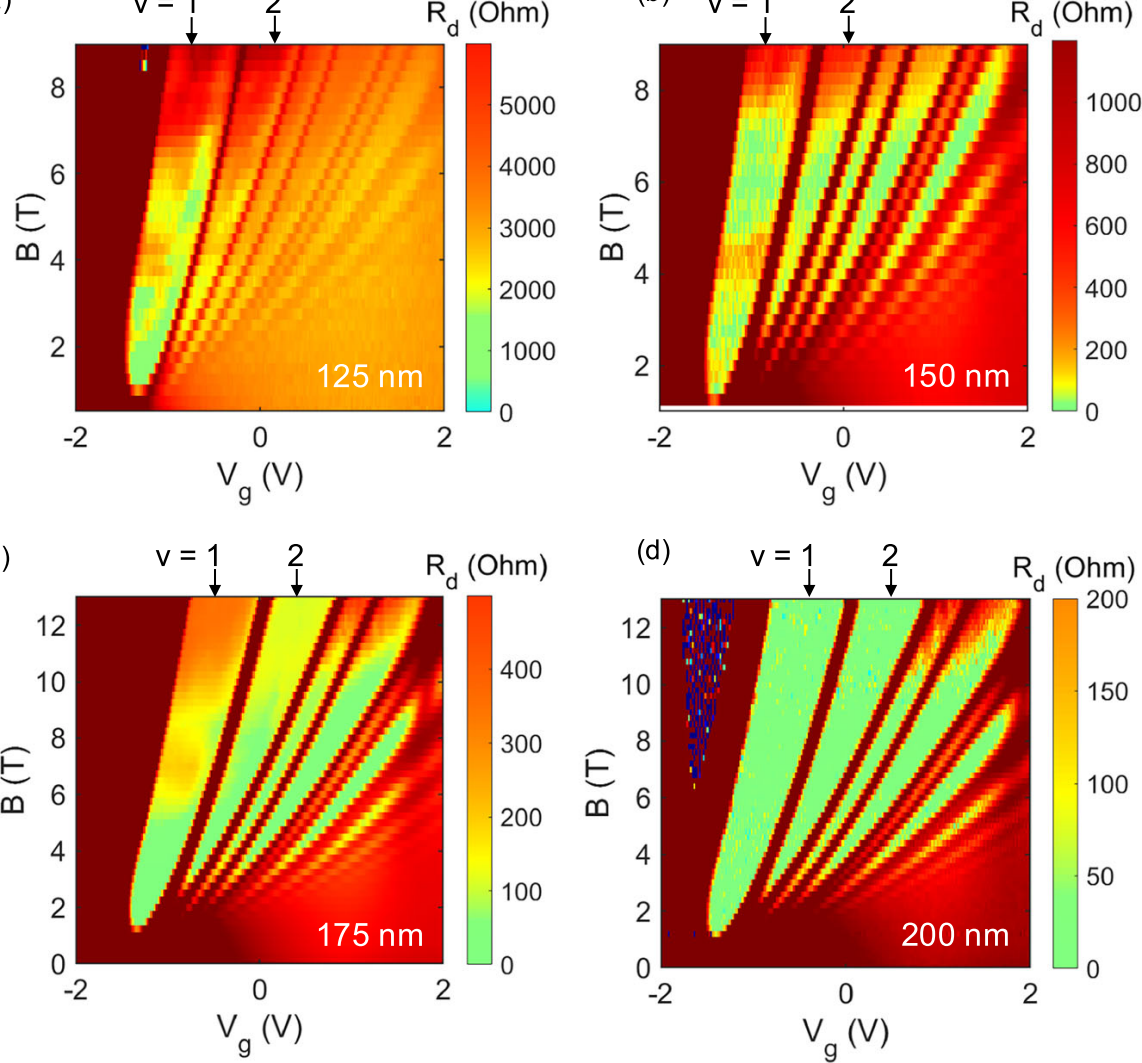
Downstream resistances of devices with different NbN finger widths. **a** 125 nm, **b** 150 nm, **c** 175 nm, and **d** 200 nm. Note that the panels have different scales for the downstream resistance *R*_d_. The peak amplitude of *R*_d_ increases with decreasing NbN width.

As shown in Fig. 1e, at high fields, the *R*_d_ maxima follow an approximate ratio of $1:\frac{1}{2}:\frac{1}{4}$ for *v* = 1, 2 and 4, respectively. According to Eq. (1), assuming that *P*_e_ and *P*_h_ are independent of *v*, ${R}_{{{\rm{d}}}}\propto \frac{1}{v}$. This suggests that the non-zero *R*_d_ originates from ECT. Regions where *R*_d_ ~ 0 may indicate that ECT and CAR are nearly equal or that both are absent. Figure 2 shows the NbN-width-dependence of *R*_d_. Oscillations in *R*_d_ become more pronounced, and the maximum values of *R*_d_ increase as the width of the NbN finger decreases. Over half of devices with 150 nm wide NbN fingers showed oscillations, while only one of the devices with a 175 nm wide finger showed discernable oscillations. For NbN widths ≥ 200 nm, *R*_d_ = *R*_*xx*_ ~ 0 at all magnetic fields. A summary of the observations for all investigated devices can be found in Supplementary Table 1.

The width dependence of the results indicates that they are caused by transmission *through* the finger. For example, *R*_d_ becomes non-zero and oscillating when the finger width is reduced by just 25 nm, which is a minor change (by almost three orders of magnitude) relative to its circumference, which appears to rule out CAES. In addition, for finger lengths that are much longer than the skipping orbits, prior studies suggest that CAES will produce a balanced ratio of electrons to holes, resulting in zero *R*_d_^16–18^. We can also rule out trivial origins, such as contact resistances or a finite resistance of the NbN finger, which would not exhibit the observed scaling with *v* (see Supplementary Note 1 for a device with a finite finger resistance).

We next briefly discuss the possible origins of the observed non-monotonic behavior of *R*_d_ in magnetic field. While omnipresent Abrikosov vortices in the NbN strips promote ECT^24^, it is not obvious how they would produce *oscillations* in magnetic fields. The number of vortices increases with increasing field, so one would expect continuous or step-wise changes of *R*_d_ in the magnetic field. Interferences from phase-coherent electron and hole-like modes in the *downstream response of CAES* have previously been studied^12,13,24,32–34^. In our devices, as discussed above, CAES are unlikely, while ECT and/or CAR by themselves cannot cause electron-hole interferences. The most likely explanation is that the transmission probabilities are non-monotonic functions of the magnetic field. A possible mechanism is an accumulation layer around the NbN finger, whose filling factor changes with the magnetic field. For example, ECT may be more likely when the accumulation layer is in a Landau level. At high magnetic fields, the Zeeman field will overwhelm any effects from intrinsic spin-orbit coupling, leading to spin-polarization of the edge states. Moreover, a very large number of vortices are present. Both favor ECT and may explain the large positive *R*_d_ above 10 T. Importantly, as long as *R*_d_ ≥ 0, any CAR contributions in the downstream modes are obscured, even if present. For this reason, we turn to thermoelectric measurements, focusing on the *v* = 1 plateau, which shows the strongest oscillations.

**Fig. 3:**
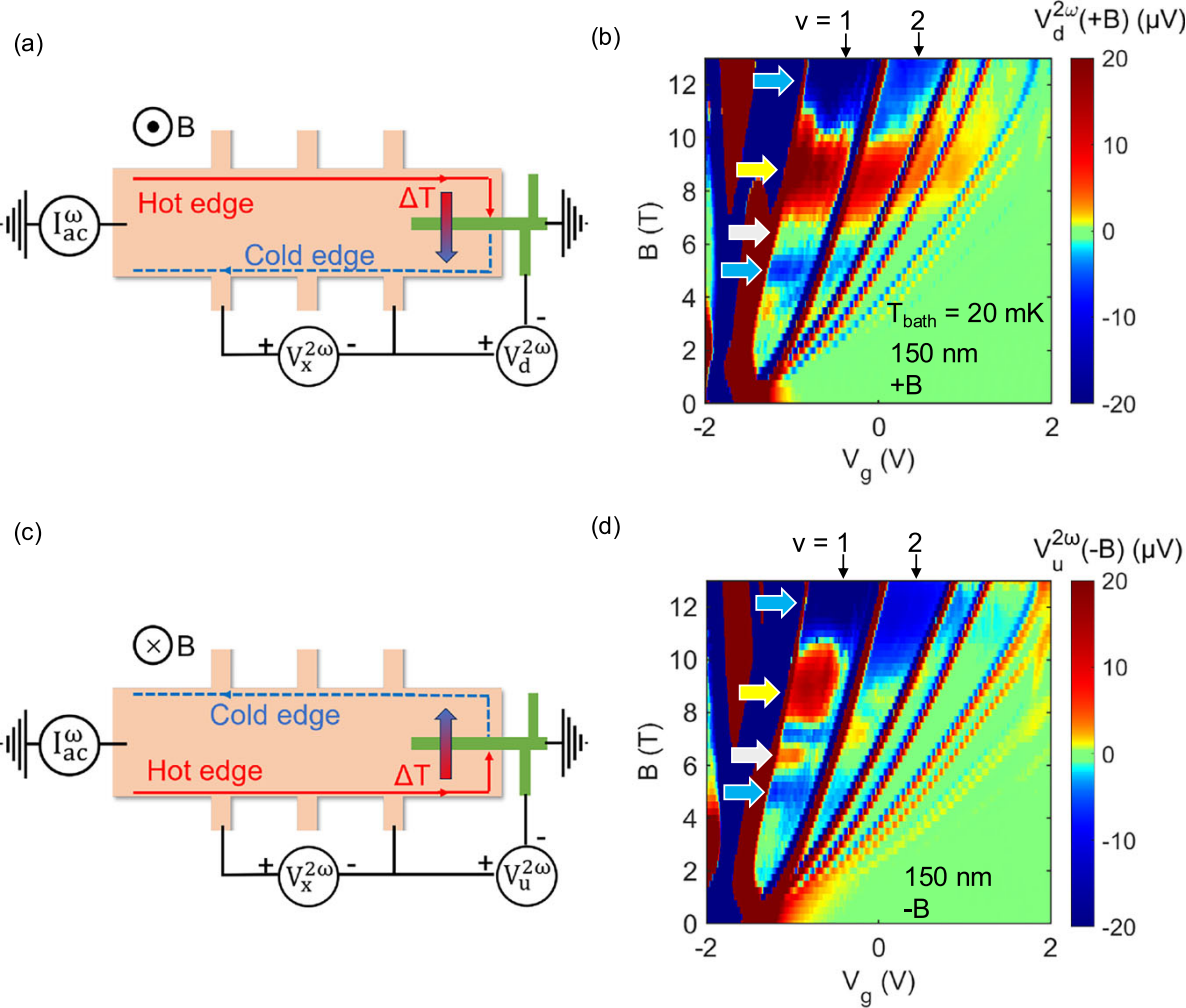
Thermoelectric measurements of Cd_3_As_2_/NbN devices. **a** Measurement setup and direction of thermal gradient (Δ*T*) for the positive magnetic fields (*B*). **b** Landau level map of the thermoelectric downstream voltage ${V}_{d}^{2\omega }\left(B\right)$ measured on the same device as Fig. 1c, d. **c** Measurement setup and direction of thermal gradient for the negative magnetic field direction. **d** Landau level map of the thermoelectric upstream voltage ${V}_{{{\rm{u}}}}^{2\omega }\left(-B\right)$ measured on the same device as in (**b**), with the voltage probes connected to the same contacts as in (**b**). Blue, yellow, and white arrows in (**c**) and (**d**) mark regions of negative, positive, and near-zero ${V}_{d}^{2\omega }$, which align with the distinct regimes in *R*_d_, shown in Fig. 1d.

To measure the downstream thermoelectric response, we use the heat-carrying properties of the chiral edge states^35–37^. A temperature difference is established by injecting hot electrons, obtained via Joule heating by large currents that are injected from the source contact, an approach that was previously developed in the literature^35,36,38^. More details of the thermoelectric measurements can be found in Supplementary Note 3. We have previously shown that in the incompressible regime, carriers in ballistic edge channels in Cd_3_As_2_ films do not cool until they equilibrate at the drain contact^37^, i.e., the superconductor. As a result, left and right moving edge channels maintain different temperatures, establishing a thermal gradient across the Hall bar and the superconducting finger and allowing for thermoelectric effects (Fig. 3a). Here we apply a 200 nA AC current at a frequency *ω* = 7.778 Hz and use lock-in techniques to measure the downstream thermoelectric voltage (denoted ${V}_{{{\rm{d}}}}^{2\omega }$ in the following) in the second harmonic (2*ω*). The 2*ω* technique ensures that ${V}_{{{\rm{d}}}}^{2\omega }$ is insensitive to the ohmic response^38,39^. The current amplitude is sufficient to create a large temperature difference, but is below the critical current of NbN in the relevant field range (Supplementary Note 4).

Figure 3b shows a Landau level map of ${V}_{{{\rm{d}}}}^{2\omega }$ recorded from the same device as in Fig. 1, while the longitudinal thermoelectric voltage (${V}_{x}^{2\omega }$) is shown in the Supplementary Note 3. Both ${V}_{x}^{2\omega }$ and ${V}_{{{\rm{d}}}}^{2\omega }$ exhibit sign-changing oscillations when the Fermi level passes through a Landau level, consistent with the theoretically expected response^37,40^. For details on the thermoelectric responses in the quantized regime, see Supplementary Note 3. Moreover, ${V}_{x}^{2\omega }$ vanishes in the plateaus, which is also consistent with theory^40,41^ (see Supplementary Fig. 3). Thus, as also discussed in more detail in the Supplementary Note 3, the devices exhibit well-behaved and well-understood thermoelectric responses in ${V}_{x}^{2\omega }$, with no unexplained contributions. In contrast, ${V}_{{{\rm{d}}}}^{2\omega }$, like *R*_d_, exhibits non-zero values within the Hall plateaus that show a non-monotonic behavior in the field. Unlike *R*_d_, however, ${V}_{{{\rm{d}}}}^{2\omega }$ exhibits sign changes and we can distinguish regions of negative and positive ${{{\rm{V}}}}_{{{\rm{d}}}}^{2{{\rm{\omega }}}}$, as well as regimes where ${V}_{{{\rm{d}}}}^{2\omega } \sim 0$.

As shown by the arrows in Figs. 1d and 3b, oscillations in ${V}_{{{\rm{d}}}}^{2\omega }$ align with those in *R*_d_. For example, the positive region of *R*_d_ near 4 T corresponds to a negative region in ${V}_{{{\rm{d}}}}^{2\omega }$, while adjacent to this region, both *R*_d_ and ${V}_{{{\rm{d}}}}^{2\omega }$ vanish. Above 7 T, ${R}_{{{\rm{d}}}}$ is positive with a step change at higher fields, at which ${V}_{{{\rm{d}}}}^{2\omega }$ switches from positive to negative. Like *R*_d_, ${V}_{{{\rm{d}}}}^{2\omega }$ remains fairly constant as a function of gate voltage, as long as the Fermi level remains within a Hall plateau, and unlike the oscillations that are seen (and expected from theory) when a Landau level is crossed. The close correspondence in the main features in *R*_d_ and ${V}_{d}^{2\omega }$ was observed in multiple devices. Data from devices with 175 nm-wide NbN fingers are presented in Supplementary Note 5. Similar to the oscillations in *R*_d_, the sign-alternating behavior of ${V}_{{{\rm{d}}}}^{2\omega }$ is absent in devices with NbN widths > 200 nm, further supporting the connection. Both types of oscillations are only observed when NbN is superconducting (Supplementary Note 6). We note that the electrical resistance measurements, including *R*d, shown in the main text were all carried out with small currents that do not cause heating, but the main features in *R*d remained unchanged even at larger currents (Supplementary Note 5). Thus, *R*_d_ is not caused by a thermoelectric effect, unlike ${V}_{{{\rm{d}}}}^{2\omega }$.

Thermoelectric measurements were also performed for the same device with the direction of the magnetic field reversed, using the same voltage probe configuration as in Fig. 3b. Reversing the field flips the chirality of the quantum Hall edge states and thus the direction of the thermal gradient^37^, see Fig. 3c. The results shown in Fig. 3d are thus the *upstream* thermoelectric voltage (${V}_{{{\rm{u}}}}^{2\omega }\left(-B\right)$). Remarkably, the main features in the *v* = 1 plateau, such as the negative ${V}_{{{\rm{u}}}}^{2\omega }\left(-B\right)$ around 5 T, positive ${V}_{{{\rm{u}}}}^{2\omega }\left(-B\right)$ near 9 T, and another negative peak beyond 10 T are similar in both upstream and downstream responses. Minor discrepancies are due to damage to the device, as multiple other high-current measurements performed before acquiring the data in Fig. 3d. In measurements on a different device (see Supplementary Note 6), which had undergone fewer measurements, the agreement between ${V}_{d}^{2\omega }\left(B\right)$ and ${V}_{u}^{2\omega }\left(-B\right)$ is nearly perfect.

We begin by discussing the origin of the nonzero values of ${V}_{{{\rm{d}}}}^{2\omega }$. The Landauer-Büttiker formalism for multi-terminal devices can be extended to include thermoelectric effects^42,43^. The current at terminal *i* is given by:

2 $${I}_{i}=\frac{{e}^{2}}{h}\mathop{\sum }\limits_{j}\left({\delta }_{{ij}}-{P}_{{ij}}\right){V}_{j}+\frac{e{\pi }^{2}{k}_{B}^{2}{T}_{0}}{3h}\mathop{\sum }\limits_{j}{\left.\frac{\partial {P}_{{ij}}}{\partial E}\right|}_{E={E}_{F}}\cdot {\theta }_{{{\rm{j}}}},$$

where *P*_*ij*_ is the transmission probability from terminal *j* to *i*, *T*_0_ is the bath temperature, *E*_*F*_ is the Fermi energy, and *θ*_*j*_ is the temperature deviation of terminal *j* from *T*_0_. We account for the thermal gradient by assigning a temperature Δ*T* to the hot edge. Using an explicit form of the scattering matrix (Supplementary Note 7), we derive an expression for ${V}_{{{\rm{d}}}}^{2\omega }$:

3 $${V}_{{{\rm{d}}}}^{2\omega }=-\frac{{\pi }^{2}{k}_{B}^{2}{T}_{0}}{3e}\cdot \frac{1}{1-{P}_{{{\rm{e}}}}+{P}_{{{\rm{h}}}}}\cdot {\left.\frac{\partial \left({P}_{{{\rm{e}}}}-{P}_{{{\rm{h}}}}\right)}{\partial E}\right|}_{E={E}_{F}}\cdot \varDelta T$$

Here, we make the assumption, shown in Fig. 3 and noted above, that the superconductor, which serves as the drain contact, remains at the bath temperature, while the thermal gradient (Δ*T*) is across the width of the superconductor. One can also imagine a scenario where the upstream side of the superconductor is heated, as was seen in ref. ^44^. In this case, Eq. (3) still applies (see Supplementary Note 7). The applicability of the extended Landauer-Büttiker formalism is confirmed by the measurements under a reversed magnetic field. In particular, the formalism predicts our finding that ${V}_{u}^{2\omega }\left(-B\right)={V}_{d}^{2\omega }\left(B\right)$ (Supplementary Note 7).

Equation (3) is a version of the Mott formula^45^ and suggests that the oscillations in *R*_d_ and ${V}_{d}^{2\omega }$ are closely aligned. Comparing Eqs. (1) and (3) we see that while measurements of *R*_d_ reflect the difference between *P*_e_ and *P*_h_, thus obscuring hole contributions unless ${P}_{e} < {P}_{h}$, the sign and magnitude of ${V}_{{{\rm{d}}}}^{2\omega }$ is controlled by differences in the energy dependence of the CAR and ECT probabilities, respectively. Although we have no information about $\frac{\partial \left({P}_{{{\rm{e}}}}\right)}{\partial E}$ and $\frac{\partial \left({P}_{{{\rm{h}}}}\right)}{\partial E}$ in our quantum Hall/superconductor system, or how they change with magnetic field, it is possible that $\frac{\partial \left({P}_{{{\rm{e}}}}\right)}{\partial E}\ne \frac{\partial \left({P}_{{{\rm{h}}}}\right)}{\partial E}$. In particular, while superconductors preserve particle-hole symmetry, the ECT/CAR probabilities are determined by the joint density of states of both the superconductor and the interfaces. For example, normal metal-superconductor-normal metal structures use quantum dots at the interface to break the particle-hole symmetry of the tunneling processes^46^. The bias-dependent data shown in the Supplementary Note 2 suggests that $\frac{\partial \left({P}_{{{\rm{e,h}}}}\right)}{\partial E}$ change with field. In particular, regions with non-zero ${V}_{d}^{2\omega }$ show a strong bias dependence of *R*_d_ ($\frac{{d}^{2}V}{\partial {I}^{2}}$ is large), indicating large changes within certain energy windows in these magnetic field regimes. In contrast, there is no such bias dependence when ${V}_{d}^{2\omega } \sim 0$. Interestingly, in both the bias dependent study (Supplementary Note 2) and in the thermoelectric experiments, the applied bias is greater than the superconducting gap of NbN. If electrons enter the superconductor with energy above the superconducting gap, no Andreev processes are expected. In these samples, the area around the superconducting finger has a higher filling factor, which may cause a potential drop before the electrons reach the interface with the superconductor. The data indicate that the interface plays an important role, as discussed above. In particular, the oscillations appear at similar magnetic fields across multiple plateaus, indicating that the bulk Hall plateau's filling factor is less important than other factors, with the interface regions the most likely candidates.

If $\frac{\partial \left({P}_{{{\rm{e}}}}\right)}{\partial E}\ne \frac{\partial \left({P}_{{{\rm{h}}}}\right)}{\partial E}$, ${V}_{{{\rm{d}}}}^{2\omega }$ can differ in sign from *R*_d_. In general, but also for tunneling, it has been shown that the sign of the thermoelectric voltage reflects the carrier type (electrons or holes)^43^. For the device shown in Figs. 1 and 3, we see that around 4 T, ${V}_{{{\rm{d}}}}^{2\omega } < 0$, while ${R}_{{{\rm{d}}}} > 0$, so both are consistent with electron-like carriers and ECT^43^. Remarkably, at high magnetic fields there is a region where ${V}_{{{\rm{d}}}}^{2\omega } > 0$ and ${R}_{{{\rm{d}}}} > 0$. We postulate that the positive ${V}_{{{\rm{d}}}}^{2\omega }$ may be explained by holes (CAR) dominating the thermoelectric response. The “filtering” properties of the thermoelectric response thus potentially allow for revealing the CAR contribution. Using thermoelectric effects was previously proposed for Cooper pair splitters that use quantum dots to tune the energy dependence of ECT and CAR^46^.

To summarize, downstream ohmic and thermoelectric responses in a Cd₃As₂/NbN quantum Hall/superconductor platform provide evidence of a competition between different types of superconducting proximity effects that can be tuned by the magnetic field. The approach can broadly be applied to other superconductor–hybrid systems. In particular, CAR are also of great interest for generating quantum entanglement via the splitting or formation of Cooper pairs^47–50^. The development of quantitative theoretical models of the observed magnetic field dependence would be very interesting.

## Methods

Devices were fabricated from an 18 nm epitaxial Cd₃As₂ film grown via molecular beam epitaxy on a (001) GaSb substrate covered with a thick Al_0.45_In_0.55_Sb buffer layer. The Cd₃As₂ thin film was capped with a 3 nm GaSb layer. Details of the growth and magnetotransport measurements were reported elsewhere^26,51^. The film was patterned into a Hall bar. Narrow NbN superconducting fingers having a length of 5 µm and widths ranging from 125 nm to 200 nm were deposited by sputtering and served as the drain contacts (defined here for electron currents). NbN was chosen for its high critical temperature (*T*_c_ ≈ 13 K) and high critical field. All NbN fingers remained superconducting to 13 T, the maximum field used here. The chemical potential was tuned by applying a top gate voltage, *V*_g_. A 20 nm Al₂O₃ layer served as the gate dielectric. The devices were measured in a dilution refrigerator with a base temperature of 20 mK. The downstream resistance *R*_d_ (Fig. 1b) was measured using lock-in amplifiers with a source current of 1 nA at a frequency of 17.778 Hz, unless specified otherwise. The thermoelectric measurements are described in the Supplementary Information.

## Supplementary information

Supplementary Information

Transparent Peer Review file

## Data Availability

The data contained in the figures of this manuscript and its supplementary information have been deposited in the Zenodo repository under the following accession link: 10.5281/zenodo.20115897.
