# Supplementary material for: Thermoelectric Detection of Crossed Andreev Reflections in Quantum Hall/Superconductor Hybrid Structures
Source: Nat Commun. 2026 Jun 5;17:7213. doi: 10.1038/s41467-026-74064-2 (PMC13396179; doi:10.1038/s41467-026-74064-2)
Supplement: Supplementary file 1 — Supplementary Information [file 41467_2026_74064_MOESM1_ESM.pdf]

Supplementary Information

Thermoelectric Detection of Crossed Andreev Reflections in Quantum Hall/Superconductor Hybrid Structures

Jiashu Wang[1], Sina Ahadi[1], Boliang Liu[1], Leon Balents[2], Simon Munyan[1], and Susanne Stemmer[1]

[1] Materials Department, University of California, Santa Barbara, California 93106-5050, USA
[2] Kavli Institute for Theoretical Physics, University of California, Santa Barbara, California 93106-4030, USA

Supplementary Note 1: Superconducting properties of the NbN fingers

The devices discussed in the main text used Ti/NbN (5 nm/70 nm) as the superconducting drain. A magnified SEM image of the finger is shown in Supplementary Fig. 1a. Wide NbN fingers had a critical temperature T_c of 13 K and an estimated upper critical field $B_{c2}(T = 0\ \mathrm{K}) > 60\ \mathrm{T}$. Both T_c and B_{c2} decrease as the width of the NbN strip is reduced. Supplementary Fig. 1b shows representative resistance traces R_{sc} of NbN fingers with different widths, measured using a lock-in amplifier connected across the two NbN leads (Supplementary Fig. 1a). Scaling of the NbN finger width was limited by the appearance of a residual resistance at ~ 100 nm (Supplementary Fig. 1c). For this reason, we only studied devices with NbN widths greater than 125 nm. The reported widths refer to the lithographic mask; the actual fabricated widths were typically 10 nm – 30 nm wider.

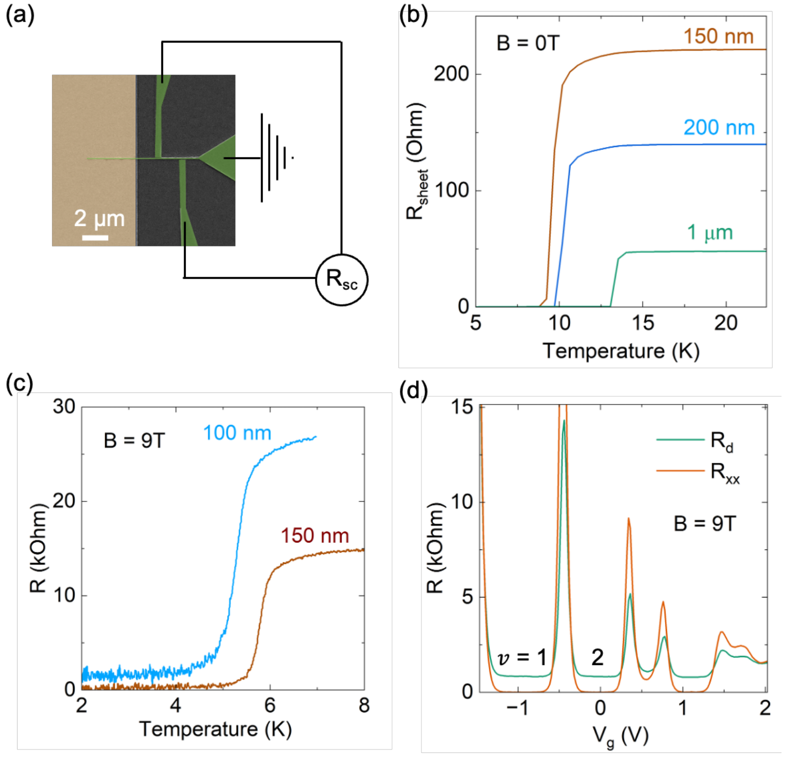

Supplementary Figure 1. Superconducting properties of the NbN fingers. (a) Schematic of the measurement setup used to characterize the resistance of the NbN fingers. (b) Suppression of T_c with decreasing NbN width. (c) Residual resistance below T_c, which limits the usable NbN width in devices. (d) Downstream resistance R_d in a device with a partially resistive NbN finger, showing a *constant* offset across all filling factors ν - distinct from the ν-dependent tunneling-induced R_d discussed in the main text.

Although R_{sc} may indicate that the fingers are superconducting, care must be taken that the NbN fingers in the final devices do not contain non-superconducting regions. In particular, areas of the NbN fingers that cover the Hall bar mesas are more prone to becoming resistive due to the NbN being thinner in these regions. Earlier devices had higher mesas and we observed an offset in R_{d} on the order of several kΩ across all filling factors. To address this issue, we modified the fabrication process by selectively etching the Cd_3As_2 layer while preserving the buffer layer. This reduces the step height at the Hall bar edges, and the offset in R_{d} was no longer observed. Supplementary Fig. 1d illustrates the behavior of R_{d} when resistive NbN contributes a background. The residual resistances at $\nu = 1, 2, 4$ does not depend on filling factor, in sharp contrast to the tunneling contributions to R_{d} discussed in the main text.

Supplementary Note 2: Differential R_{d} under an applied bias

To further investigate the origin of the peaks in R_{d}, a DC bias voltage was swept while maintaining a 1 nA AC current under constant gate voltage V_{g} and magnetic field. The R_{d} measured in this configuration corresponds to a differential resistance as a function of DC bias. Results for selected magnetic fields and Hall plateaus are shown in Supplementary Fig. 2. Notably, within magnetic field ranges where R_{d} shows positive peaks, we observe a pronounced DC bias dependence. Further increasing the DC bias caused a suppression of R_{d}. In contrast, when $R_{\mathrm{d}} \sim 0$, the differential resistance is bias independent, until it becomes large enough to suppress superconductivity.

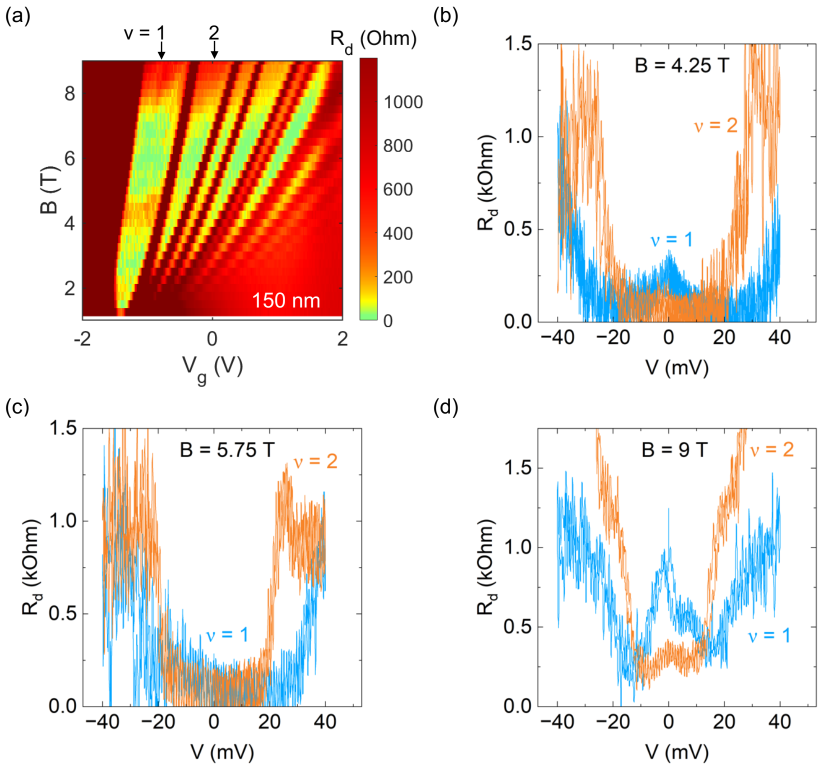

Supplementary Figure 2. Measurements of R_{d} with an additional DC voltage bias. (a) Map of R_d measured in a 150 nm-wide device, same device as in Fig. 2b. (b-d) Differential resistance R_d measured as a function of applied DC bias voltage with fixed V_{g} and magnetic field within the $\nu =$ 1 and $\nu = 2$ plateaus, respectively. At magnetic fields where R_{d} shows a maximum, R_{d} is strongly bias dependent.

The measurements indicate that the NbN remains superconducting within the magnetic field range used in this study. A clear transition to the normal state is observed at high DC bias. For example, in Supplementary Fig. 2d at 9T, R_d undergoes a superconducting transition near 20 mV, corresponding to a critical current of $I_c = \frac{V_\mathrm{dc}}{h/e^2} \approx 770$ nA. This is significantly higher than the current amplitudes used in both our first-order (1 nA) and second-harmonic (200 nA) measurements.

An interesting question raising from this experiment is that the applied voltage bias is larger than the superconducting gap of NbN (< 1 meV). Similarly, at $\nu = 1$ and a current of 200 nA (used for the thermoelectric experiments), the voltage bias is about 5 meV. A possible explanation is that the applied voltage does not correspond to the actual potential at the NbN/Cd_3As_2 interface, but that part of the voltage drops before electrons reach the superconducting interface. In particular, experiments indicate that NbN causes an accumulation layer in the surrounding Cd_3As_2, resulting in a higher effective filling factor ν'. If this accumulation layer is not fully proximitized, a partial voltage drop can occur prior to any Andreev or tunneling processes. Since $\nu' > \nu$, this effect is not directly reflected in the total resistance.

Supplementary Note 3: Details of the thermoelectric measurements

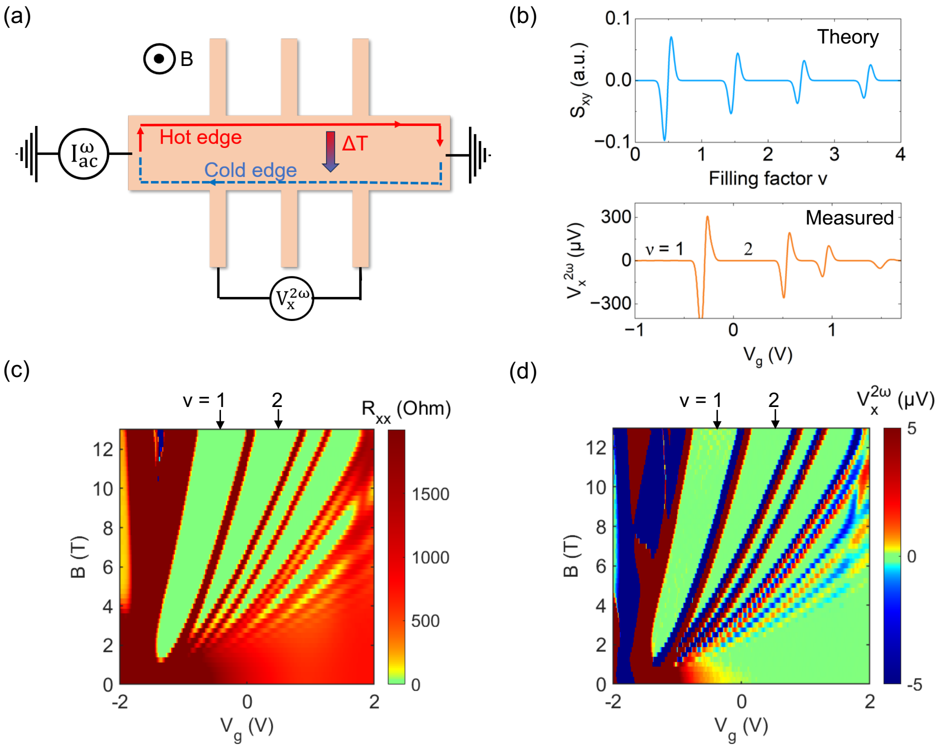

Supplementary Figure 3. Thermoelectric measurements. (a) Open-circuit measurement setup. (b) Theoretically predicted behavior of S_{xy} and measured $V_x^{2\omega}$. (c). (d,e) Landau level maps of R_{xx} and $V_x^{2\omega}$.

A thermal gradient is generated by injection of hot electrons into the edge channels, see e.g., refs. (*1*-*3*). A large current I injected at the source heats the contact with power $P = I^2R = \frac{I^2h}{\nu_b e^2}$, where R is the interface resistance, determined by the bulk filling factor ν_b. The edge mode leaves the source with chemical potential $\mu = I\frac{h}{\nu_\mathrm{b} e^2}$ and electron-electron scattering quickly thermalizes the modes into a hot Fermi-Dirac distribution. As indicated in Fig. 3a and Supplementary Fig. 3a, the high potential edge establishes the hot side of the device. Reversing the direction of the field (Fig. 3c) reverses which side is hot, due to the chirality of the edge states.

Hot electrons in ballistic, chiral edge states on one edge maintain their temperature until they encounter the cold drain, where they equilibrate. The counterpropagating edge states on the opposite edge, which flow in the reverse direction, maintain the temperature of the cold drain. This establishes a temperature gradient across the Hall bar, see Fig. 3 and Supplementary Fig. 3a. The thermoelectric measurements were carried out using the 2ω method that is widely used, see e.g., refs. (*1, 4*) for prior work. We briefly describe the technique here. To generate hot carriers, a large AC current (200 nA) with frequency ω (7.778 Hz in our experiments) is applied. The thermoelectric voltage oscillates at 2ω, since:

$$V^{2\omega} \propto \Delta T \propto I^2 \sin^2(\omega t) = \frac{I^2}{2}\,[1 - \cos(2\omega t)] \tag{1}$$

Using lock-in techniques, the thermoelectric voltage is measured as the second harmonic 2ω. Because the temperature gradient in our experiments is along the *y*-direction, $V_x^{2\omega}$ is a Nernst voltage. In general:

$$V_x^{2\omega} = S_{xx}\Delta T_x + S_{xy}\Delta T_y, \tag{2}$$

where ΔT_x and ΔT_y are the temperature gradients along the *x*- and *y*-directions, respectively, S_{xx} is the thermopower (Seebeck coefficient) and S_{xy} is the Nernst coefficient. Since $\Delta T_x \sim 0$:

$$V_x^{2\omega} = S_{xy}\Delta T_y. \tag{3}$$

Theory by Jonson and Girvin (*5*) gives the expressions for S_{xy} in the quantized regime, where $\rho_{xx} < \rho_{xy}$:

$$S_{xy} \approx -\rho_{xy}\alpha_{xx}, \tag{4}$$

where ρ_{xy} is the Hall resistivity and α_{xx} is the diagonal element of the thermoelectric tensor. Based on the model, $S_{xy} = -\frac{\pi^2 k_B^2 T}{3e\sigma}\frac{\partial\sigma}{\partial\mu}$ follows the Mott relation, resulting in sign-oscillations when crossing Landau levels. Supplementary Fig. 3b shows theoretically calculated and measured behavior of S_{xy} and $V_x^{2\omega}$, respectively. A detailed discussion on the $V_x^{2\omega}$ without superconductivity can also be found in ref. (*6*). Note the good agreement between experiment and theory, including the oscillations of S_{xy} and $V_x^{2\omega}$ when the Fermi level traverses a Landau level and the zero values at the Hall plateaus. Supplementary Figs. 3c and 3d show Landau level maps of R_{xx} and $V_x^{2\omega}$, showing excellent correspondence. Note again that $V_x^{2\omega}$ oscillates at the Landau levels and is zero at the Hall plateaus. The downstream thermoelectric voltage, $V_{\mathrm{d}}^{2\omega}$, like R_{d}, behaves differently, as discussed in the main text.

Supplementary Note 4: Current dependence of the thermoelectric measurements

We investigated the dependence of the thermoelectric signal on the AC current. The thermoelectric voltage follows a parabolic relationship with the applied current, as $V^{2\omega} \propto \Delta T \propto I^2$. If the currents are too large, this may cause heating of the superconductor and eventually cause the superconducting finger to reach the normal state. Moreover, carriers may have higher energies than the superconducting gap, as discussed above. Both will reduce the tunneling probability and, consequently, the downstream thermoelectric voltage. Supplementary Fig. 4 shows the behavior of R_{d} and $V_{\mathrm{d}}^{2\omega}$ in the $\nu = 1$ plateau at a magnetic field value where R_{d} shows a large positive peak. $V_{\mathrm{d}}^{2\omega}$ initially increases with current following a parabolic trend, consistent with an increase in the thermal gradient. $V_{\mathrm{d}}^{2\omega}$ begins to decrease above ~600 nA. From the corresponding R_{d} value, we estimate the critical current to be just above 1 μA, explaining the decrease of $V_{\mathrm{d}}^{2\omega}$. To prevent damaging the device, we did not apply currents beyond this point.

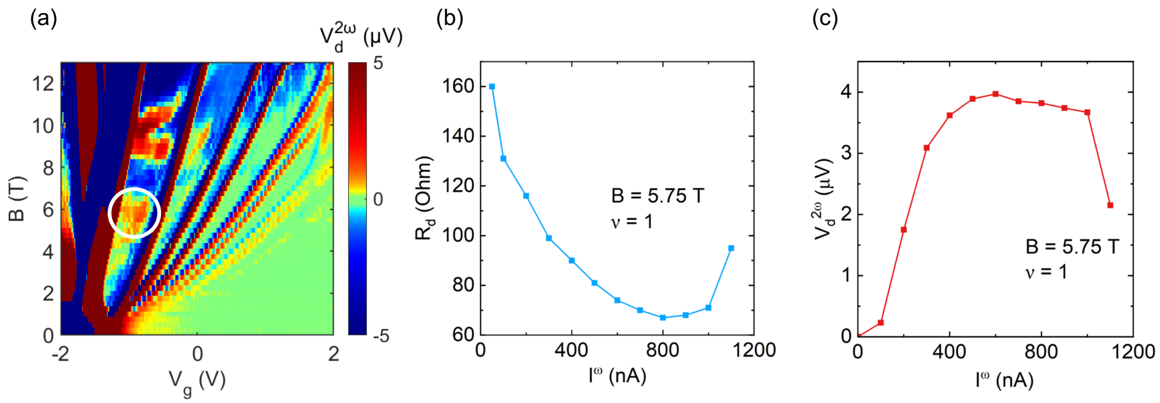

Supplementary Figure 4. AC current dependence of $V_d^{2\omega}$. (a) $V_d^{2\omega}$ map taken at a current of 200 nA. The white circle marks the experimental parameters used for the current-dependence measurements shown in (b) and (c). (b) R_d as a function of current. (c) $V_d^{2\omega}$ as a function of AC current. The signal first increases quadratically with current, consistent with Joule heating, but is suppressed when $I \gtrsim 400$ nA.

Supplementary Note 5: Additional thermoelectric data

In the main text, the similarities in the magnetic-field dependent oscillations in R_d and $V_d^{2\omega}$ were discussed. Supplementary Fig. 5 shows good agreement in the R_d maps that were recorded with the same current (200 nA) as the thermoelectric measurements. In Supplementary Fig. 6 we present additional data to show that the magnetic-field dependent oscillations are found in many different devices. These measurements were taken under the same conditions as those on the device discussed in the main text. We show examples of devices with strong oscillations, weaker ones and one without. Only the device with strongest oscillations shows sign reversals in $V_d^{2\omega}$. We note that these devices had 175 nm-wide NbN fingers. Generally, at this width, which is close to the critical width of 200 nm, the oscillations became weaker and fewer devices showed oscillations. They completely vanish in the devices with 200 nm-wide fingers (see discussion in the main text). A list of all samples and a summary of observations can be found in Supplementary Note 9.

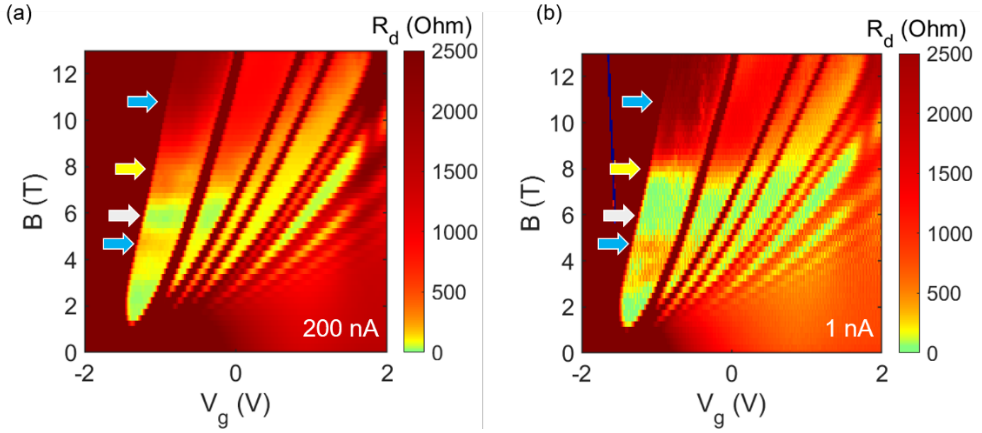

Supplementary Figure 5. (a) Landau level map of R_d recorded with the same current density (200 nA) as the thermoelectric data shown in Fig. 3. Regimes of different R_d values remain at the same position as the map measured with 1 nA, shown for comparison in (b). The magnitude of the R_d peaks is slightly lower, due to the higher bias, as expected from Supplementary Fig. 2.

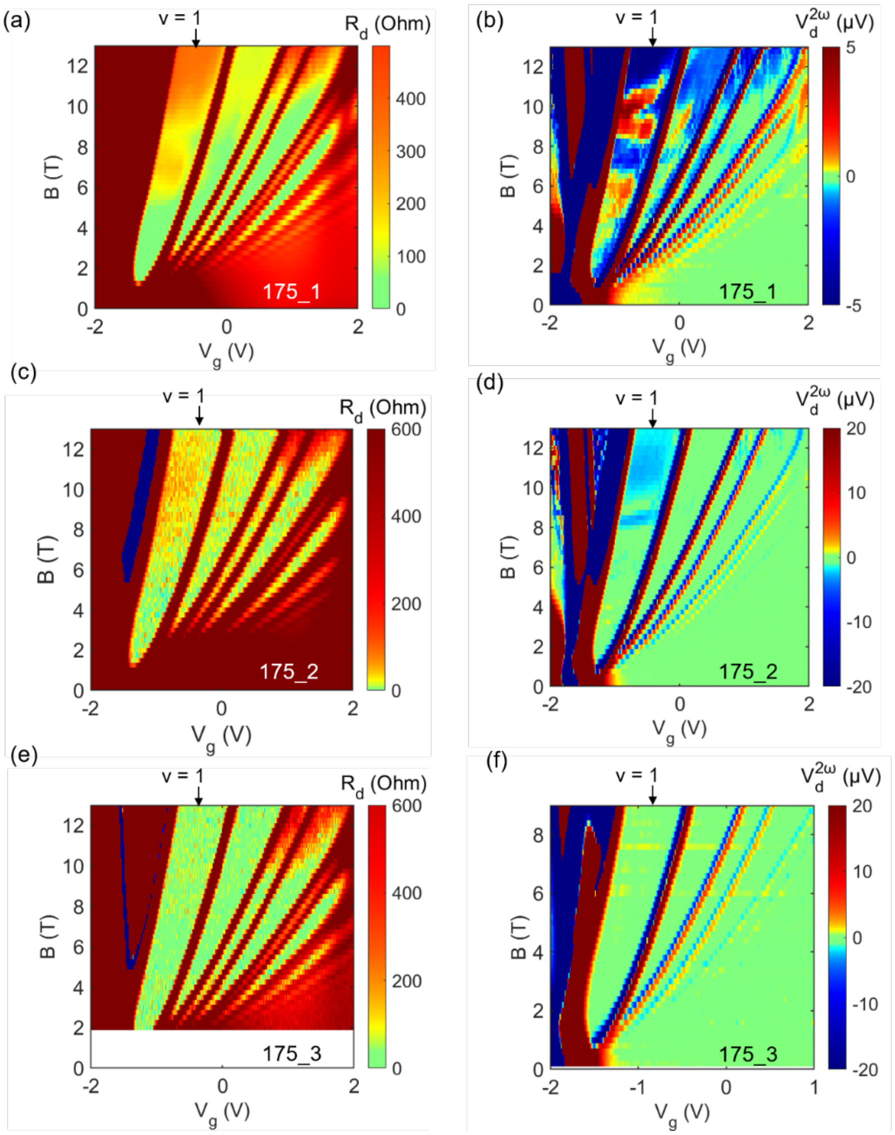

Supplementary Figure 6. R_d (right column) and $V_\mathrm{d}^{2\omega}$ (left column) Landau maps for three additional devices with 175 nm-wide NbN fingers.

Supplementary Note 6: Extended Landauer-Büttiker formalism

The current between a two-terminal device in the ballistic limit can be described using the Landauer-Büttiker formalism (*7, 8*):

$$I = \frac{e}{\pi\hbar}\int_{-\infty}^{+\infty} P(E)[f_1(E+V, T+\Delta T) - f_2(E,T)]\,dE, \qquad (5)$$

where $P(E)$ is the transmission probability and $f_{1,2}(E,T)$ is the Fermi distribution of the two terminals. When $P(E)$ is a function of energy, a temperature difference ΔT causes a difference in the Fermi distributions and drives a thermoelectric current (*8*):

$$I = -\frac{e^2}{h}P(E_F)V + \frac{e\pi^2 k_B^2 T}{3h}\left.\frac{\partial P(E)}{\partial E}\right|_{E=E_F}\Delta\mathrm{T} \qquad (6)$$

Equation 6 is an extension of the standard transport formula $I = -\frac{e^2}{h}PV$. Similarly, the Landauer-Büttiker formalism for multi-terminal devices, $I = \boldsymbol{G}V$, can also be extended to $I = \boldsymbol{G}V + \boldsymbol{L}\theta$, with the second term corresponding to the thermoelectric contribution (*7*):

$$I_i = \frac{e^2}{h}\sum_j\left(\delta_{ij} - P_{ij}\right)V_j + \frac{e\pi^2 k_B^2 T_0}{3h}\ \sum_j \frac{\partial P_{ij}}{\partial E}\cdot \theta_j, \tag{7}$$

where I_i is the current flowing into contact i, P_{ij} is the transmission probability, T_0 is the base temperature, θ_j is the temperature difference between the bath temperature and the *j*-th terminal. The filling factor v is set to be 1. We consider a four-terminal device sketched in Supplementary Fig. 7, where contact 1 is the source and contact 3 is the superconductor drain. As discussed in the main text, the upstream edge states are hotter than the bath temperature. While the voltage contacts remain cold, we treat voltage probes to represent the nearby spot of the edge state so we can calculate thermal voltages based on the temperature gradient. We let $\theta_1 = \theta_2 = \Delta T^{0\omega} + \Delta T^{2\omega}$, corresponding to the constant and second-order term in $I^2\sin^2(\omega t) = I^2/2\ (1 - \cos\ (2\omega t)$. The matrix can be explicitly written out:

$$\begin{pmatrix} I^\omega \\ 0 \\ -I^\omega \\ 0 \end{pmatrix} = \frac{e^2}{h}\begin{pmatrix} 1 & 0 & 0 & -1 \\ -1 & 1 & 0 & 0 \\ 0 & -(1-P_e+P_h) & (1-P_e+P_h) & 0 \\ 0 & -(P_e-P_h) & -(1-P_e+P_h) & 1 \end{pmatrix}\begin{pmatrix} V_1^{\omega,2\omega} \\ V_2^{\omega,2\omega} \\ V_3 = 0 \\ V_4^{\omega,2\omega} \end{pmatrix} +$$

$$\frac{e\pi^2 k_B^2 T_0}{3h}\begin{pmatrix} 0 & 0 & 0 & 0 \\ 0 & 0 & 0 & 0 \\ 0 & -\partial(P_e-P_h)/\partial E & \partial(P_e-P_h)/\partial E & 0 \\ 0 & \partial(P_e-P_h)/\partial E & -\partial(P_e-P_h)/\partial E & 0 \end{pmatrix}\begin{pmatrix} \Delta T^{0\omega} + \Delta T^{2\omega} \\ \Delta T^{0\omega} + \Delta T^{2\omega} \\ 0 \\ 0 \end{pmatrix} \tag{8}$$

For the ohmic response, we obtain the classical expression for the downstream voltages:

$$V_1^\omega = V_2^\omega = \frac{1}{1-P_e+P_h}\frac{h}{e^2}\cdot I \tag{9}$$

$$V_4^\omega = \frac{P_e-P_h}{1-P_e+P_h}\frac{h}{e^2}\cdot I \tag{10}$$

For the second harmonic, with $I^{2\omega} = 0$, we obtain:

$$V_1^{2\omega} = V_2^{2\omega} = V_4^{2\omega} = -\frac{\pi^2 k_B^2 T}{3e}\frac{1}{1-P_e+P_h}\frac{\partial(P_e-P_h)}{\partial E}\bigg|_{E=E_F}\cdot \Delta T^{2\omega} \tag{S11}$$

Equation 11 shows that the upstream thermoelectric voltage $V_u^{2\omega}$ is the same as the downstream thermoelectric voltage $V_d^{2\omega}$, as seen in the data discussed in the main text. Supplementary Figs. 7b and 7c show another data set comparing $V_d^{2\omega}(B)$ and $V_u^{2\omega}(-B)$. The main features between two maps agree well. The downstream and upstream resistances change by the same magnitude due to ECT or CAR. When there is no tunneling through the superconductor ($P_e = P_h = 0$), all thermoelectric voltages vanish.

The approach can be expanded to consider additional experimental conditions. For example, the incoming hot edge states could locally heat the top interface of the superconductor, as the superconductor does not conduct heat well. In this scenario, we let contact 2 represent this hot top edge. Since the transmission probability remains a constant between any contact along the upstream edge, all voltage probes along the upstream edge shares the same voltage with contact 2. We can set temperature of 2 to be different from 1, such that $\theta_{1,2} = \Delta T^{0\omega} + \Delta T_{1,2}^{2\omega}$. The superconductor itself (bottom edge) could also have a different temperature $\Delta T_3^{2\omega}$. The matrix (Equation 8) becomes:

$$\begin{pmatrix} I^{\omega} \\ 0 \\ -I^{\omega} \\ 0 \end{pmatrix} = \frac{e^2}{h}\begin{pmatrix} 1 & 0 & 0 & -1 \\ -1 & 1 & 0 & 0 \\ 0 & -(1-P_e+P_h) & (1-P_e+P_h) & 0 \\ 0 & -(P_e-P_h) & -(1-P_e+P_h) & 1 \end{pmatrix}\begin{pmatrix} V_1^{\omega,2\omega} \\ V_2^{\omega,2\omega} \\ V_3=0 \\ V_4^{\omega,2\omega} \end{pmatrix} + \frac{e\pi^2 k_B^2 T_0}{3h}\begin{pmatrix} 0 & 0 & 0 & 0 \\ 0 & 0 & 0 & 0 \\ 0 & -\partial(P_e-P_h)/\partial E & \partial(P_e-P_h)/\partial E & 0 \\ 0 & \partial(P_e-P_h)/\partial E & -\partial(P_e-P_h)/\partial E & 0 \end{pmatrix}\begin{pmatrix} \Delta T_1^{0\omega}+\Delta T_1^{2\omega} \\ \Delta T_2^{0\omega}+\Delta T_2^{2\omega} \\ \Delta T_3^{0\omega}+\Delta T_3^{2\omega} \\ 0 \end{pmatrix} \tag{12}$$

The result is similar and a second-harmonic voltage is generated from the temperature gradient across the superconductor:

$$V_1^{2\omega} = V_2^{2\omega} = V_4^{2\omega} = -\frac{\pi^2 k_B^2 T}{3e}\frac{1}{1-P_e+P_h}\frac{\partial(P_e-P_h)}{\partial E}\bigg|_{E=E_F} \cdot (\Delta T_2^{2\omega} - \Delta T_3^{2\omega}) \tag{13}$$

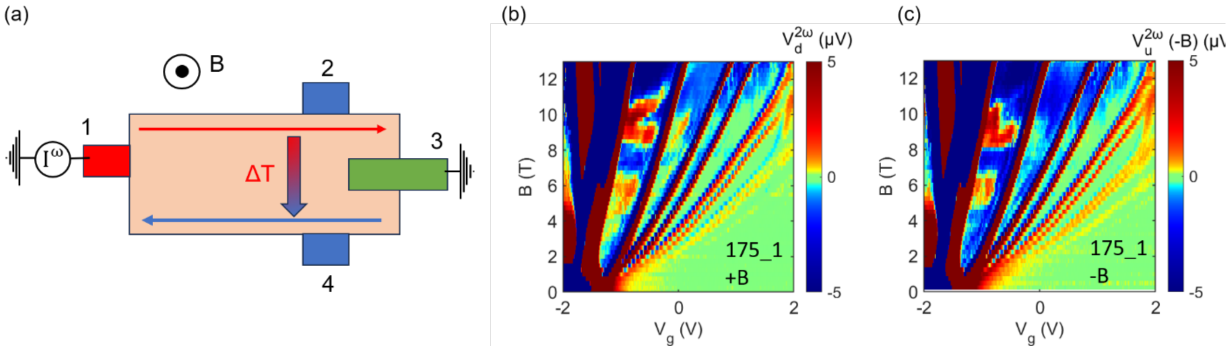

Supplementary Figure 7. (a) Sketch of a multi-terminal device. Contact 1 is the current source and contact 3 is the superconducting drain. Hot electrons are injected into the edge channel from contact 1 and the edge channel equilibrates at contact 3. (b, c) Landau level maps of $V_d^{2\omega}(B)$ and $V_u^{2\omega}(-B)$ measured on the same device as the one measured in the top row of Supplementary Fig. 6.

Supplementary Note 7: Temperature dependence of R_d and $V_d^{2\omega}$

We performed temperature-dependent measurements on the 150 nm device discussed in the main text. The critical temperature for the 150 nm NbN finger is ~10 K (Supplementary Fig. 1b). Supplementary Figs. 8a–c show R_d maps at 2 K, 4 K and 10 K, on the same scale as Fig. 2b, to allow direct comparison of oscillation strength. A major difficulty is that the quantum Hall plateaus are not well quantized above 5 K. As a result, the plateaus are narrow and assume non-zero values with increasing temperature. The oscillations are weaker at 4 K and become unobservable at 10 K. Note that at 10 K, an offset due to the normal resistance of NbN must be subtracted from R_d.

We also measured $V_d^{2\omega}$ at 10 K, as shown in Supplementary Fig. 8d. The poor quantization at 10 K is also reflected in the $V_d^{2\omega}$ maps: oscillations at the Landau levels become very broad and the plateau values are influenced by the tails of the Landau levels. The absence of the field-dependent oscillations in both R_d and $V_d^{2\omega}$ at 10 K, when the superconductivity in NbN vanishes, supports the claim that the non-zero values of R_d and $V_d^{2\omega}$ in the plateaus originate from Andreev or tunneling processes.

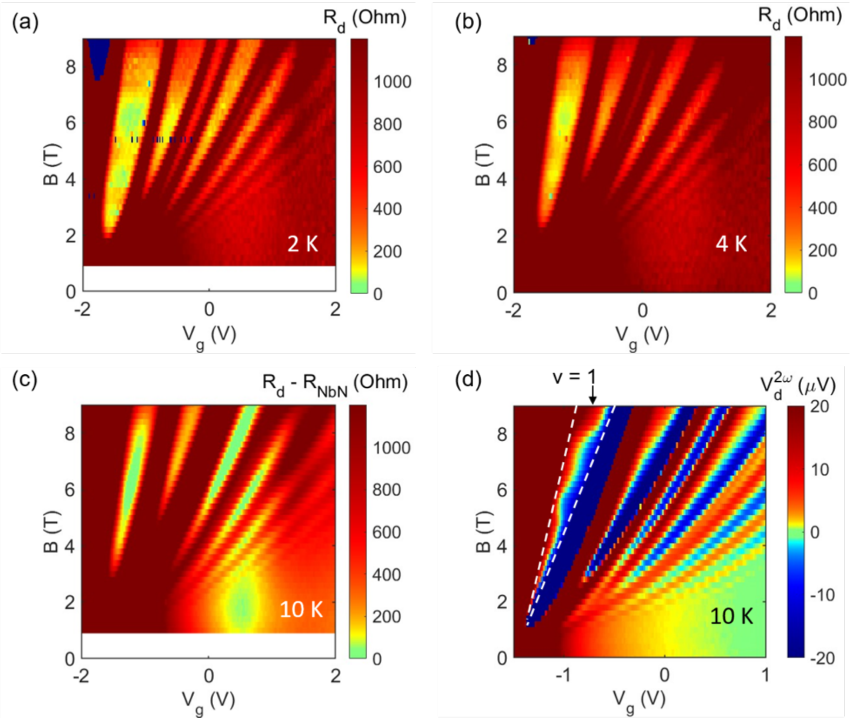

Supplementary Figure 8. R_d map measured below T_c at (a) 2 K (b) 4 K and above T_c at (c) 10 K. An offset is applied to (c) to account for NbN normal resistance. (d) $V_d^{2\omega}$ measured at 10 K. At this temperature LLs becomes wider due to the loss of good quantization. Two white dashed lines mark the region of $v = 1$.

Supplementary Note 8: Interface transparency of NbN/Cd_3As_2

It is difficult to directly assess the interface transparency of superconducting/quantum Hall devices. To investigate the interface transparency, we fabricated Josephson junctions. The device geometry is shown in Supplementary Fig. 9a. Two T-shaped NbN contacts the superconducting contacts. The spacing between them is 300 nm. The deposition process of NbN is the same as for the Hall bar fingers, but the NbN thickness is lower, due to constraints of the lift-off process (25 nm instead of 70 nm).

Measurements were carried out in a dilution refrigerator with a base temperature of 20 mK. An AC excitation current I_{ac} of 10 nA was supplied with an additional DC current bias I_{dc}. Supplementary Fig. 9b shows the differential resistance, dV/dI, as a function of I_{dc} for junctions with NbN contact widths (t). All junctions are superconducting at zero I_{dc}. As I_{dc} increases, the junctions go normal as seen by the jump in dV/dI. The normal state resistance R_N is given by the dV/dI value at high bias. Note that only the junctions become normal, while the NbN fingers remain superconducting. These measurements allow us to determine the excess current I_{ex}, defined as:

$$I_{ex} = I_{sc} - I_N|_{eV \gg \Delta}, \tag{14}$$

where I_{sc} is the current through the junction and I_N is the theoretical current in the normal state. A measure of the interface transparency τ can be obtained from the relationship $\tau = I_{ex}R_N/\Delta$. We rewrite the excess current in the voltage form:

$$V = I_{ex}R_N = V_{sc} - V_N \tag{15}$$

In Supplementary Fig. 9c we show V_{sc} and V_N as a function of I_{dc}. V_{sc} is calculated by integration of dV/dI and $V_N = I_{dc} \cdot R_N$. The difference between the solid and dashed lines is $V|_{t=180} = 82$ µeV and $V|_{t=260} = 64$ µeV.

The superconducting gap Δ of the proximitized area is determined by measuring critical temperature T_c, as shown in Supplementary Fig. 9d. For both types of junctions, the superconducting gap is estimated to be $\Delta \approx 1.76 k_{\mathrm{B}} T_{\mathrm{c}} = 0.3$ meV. The interface transparencies are estimated to be $\tau|_{t=180} = 0.27$ and $\tau|_{t=260} = 0.21$. In Supplementary Fig. 9e we show a map of the junction as a function of magnetic field and current bias. The occurrence of a nearly-ideal Fraunhofer pattern indicates a good quality junction.

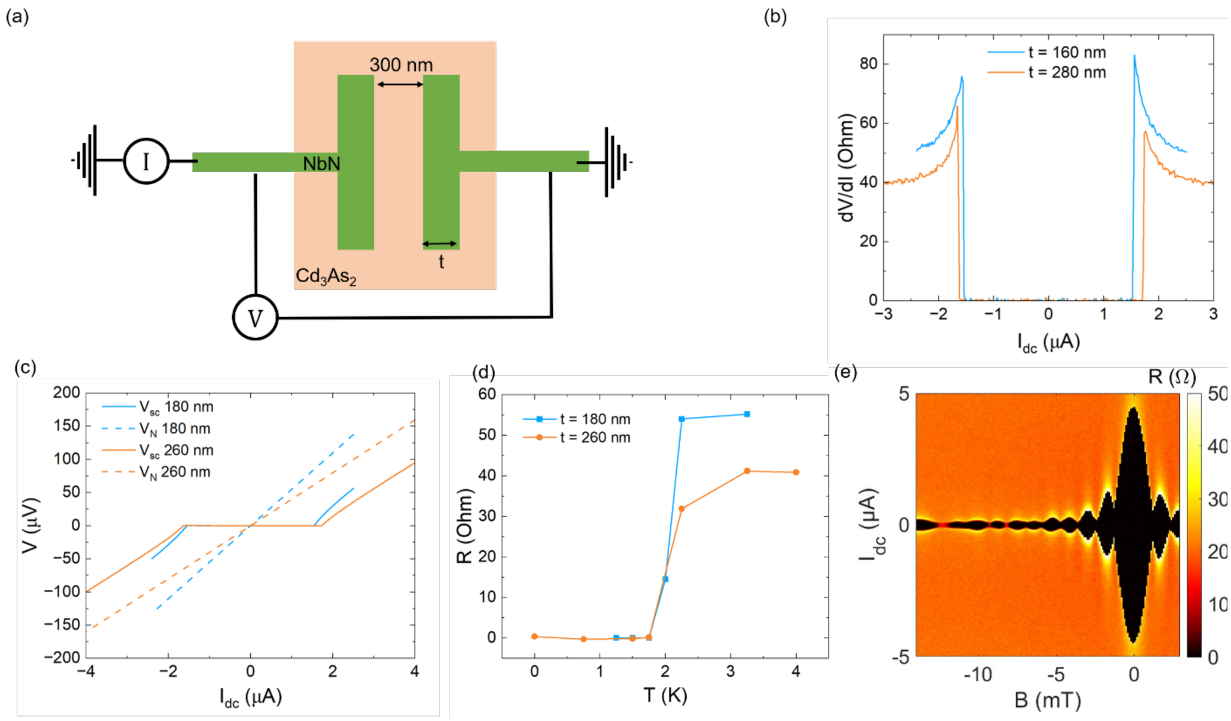

Supplementary Figure 9. Interface transparency of Josephson junctions. (a) Sketch of the junction made with NbN (green) and Cd_3As_2 (orange). (b) Differential resistance as a function of DC current bias. (c) Integrated DC voltage and a normal-resistance fit based on bias measurement shown in (b). (d) Temperature-dependent measurement of the junction resistance. (e) Fraunhofer interference pattern of the junction.

Supplementary Note 9: Sample list

Supplementary Table 1 lists the sample studied in this investigation. Also provided are the NbN finger width and whether oscillations in R_{d} and sign-reversals in $V_d^{2\omega}$ were observed. In general, effects are stronger in devices with 150 nm wide fingers compared to devices with wider fingers. For the device with 125 nm NbN, the superconducting finger has finite resistance at 200 nA and high magnetic field and it was not studied in further detail.

Supplementary Table 1. Device list and main features (see main text). Data from devices marked in blue are shown in the main text. Data from devices 175_1 to 175_3 (marked in green) are shown in the Supplementary Fig. 6.

**Device ID**	**NbN width (nm)**	**Oscillations in R_d?**	**Sign-reversal in $V_d^{2\omega}$?**
125_1	125	Y*	Y
150_1	150	N	N
150_2	150	Y	Y
150_3	150	Y	Y
150_4	150	N	N
150_1B	150	Y	Y
150_2B	150	Y	Y
175_1	175	Y	Y
175_2	175	N, only a weak peak at high field	N, only a negative signal at high field
175_3	175	N	N
200_1	200	N	Not measured
200_2	200	N	N
200_3	200	N	N

* The 125 nm device had a finite NbN resistance. For this reason, we did not perform further studies on this device.

Supplementary References

1. W. E. Chickering, J. P. Eisenstein, J. L. Reno, Hot-Electron Thermocouple and the Diffusion Thermopower of Two-Dimensional Electrons in GaAs. *Phys. Rev. Lett.* **103**, 046807 (2009).
2. G. Granger, J. P. Eisenstein, J. L. Reno, Observation of Chiral Heat Transport in the Quantum Hall Regime. *Phys. Rev. Lett.* **102**, 086803 (2009).
3. S.-G. Nam, E. H. Hwang, H.-J. Lee, Thermoelectric Detection of Chiral Heat Transport in Graphene in the Quantum Hall Regime. *Phys. Rev. Lett.* **110**, 226801 (2013).
4. S. Faniel *et al.*, Thermopower of Interacting GaAs Bilayer Hole Systems in the Reentrant Insulating Phase Near ν =1. *Phys. Rev. Lett.* **94**, 046802 (2005).
5. M. Jonson, S. M. Girvin, Thermoelectric effect in a weakly disordered inversion layer subject to a quantizing magnetic field. *Phys. Rev. B* **29**, 1939-1946 (1984).
6. W. Huynh, B. Liu, S. Munyan, S. Ahadi, S. Stemmer, Chiral edge state control of thermoelectric effects. *Sci. Adv.* **11**, eady9006 (2025).
7. P. N. Butcher, Thermal and electrical transport formalism for electronic microstructures with many terminals. *J. Phys.: Condens. Matter* **2**, 4869-4878 (1990).
8. M. Paulsson, S. Datta, Thermoelectric effect in molecular electronics. *Phys. Rev. B* **67**, 241403 (2003).
