# Supplementary material for: Thermoelectric Detection of Crossed Andreev Reflections in Quantum Hall/Superconductor Hybrid Structures
Source: Nat Commun. 2026 Jun 5;17:7213. doi: 10.1038/s41467-026-74064-2 (PMC13396179; doi:10.1038/s41467-026-74064-2)
Supplement: Supplementary file 2 — Transparent Peer Review file [file 41467_2026_74064_MOESM2_ESM.pdf]

nature portfolio

Peer Review File

Thermoelectric Detection of Crossed Andreev Reflections in Quantum Hall/Superconductor Hybrid Structures

Corresponding Author: Professor Susanne Stemmer

This file contains all reviewer reports in order by version, followed by all author rebuttals in order by version.

Attachments originally included by the reviewers as part of their assessment can be found at the end of this file.

Version 0:

Reviewer comments:

Reviewer #1

(Remarks to the Author)
Please see attached pdf.

Reviewer #2

(Remarks to the Author)
The authors present experimental results on the electric and thermoelectric transport in edge quantum Hall edge modes of cadmium arsenide with a thin superconducting NbN strip. They find downstream resistance oscillation as a function of the magnetic field, indicative of the competition between elastic cotunneling (ECT) and cross Andreev reflection (CAR). They measure the thermoelectric response and identify signatures of hole-like carriers.
The topic is very interesting and timely. The experimental results are very carefully presented and analyzed. I believe that the present contribution has the potential for being published in Nature Communication. However, before recommending its publication I suggest the Authors to revise the manuscript and carefully address the following points:
Questions:
1. Fig. 1 a: Please check the edge state injected from V_d and propagating to the left. I think it should be drawn as a solid blue line.
2. I suggest indicating the temperature in the Figs, although it is reported in the section “Methods”.
3. Before Eq. 1, the Authors state that “in the absence of CAR and/or ECT R_d equals the longitudinal resistance R_{xx}”. Is this also true in the presence of chiral Andreev states (CAES) circulating along all the length of width of the finger? If the dimensions of the finger rule out the existence of such states, the Authors should mention and discuss this.
4. In Page 7 it is mentioned that at high magnetic fields, spin polarization of the edge states makes CAR difficult. This is true. However, they mentioned in the introduction that these films have a large spin-orbit and this could change their conclusion [see PRB 83, 195441 (2011), Ref. 28 of the present manuscript and PRB 109, 024504 (2012)].
5. Fig.3. The features observed within the $\nu=2$ and higher filling factors look quite different in panels (b) and (d). Do the Authors have an explanation for this behavior? The agreement seems to be better for the results presented in Fig. S7.
6. In order to have a thermoelectric response, it is necessary to have a particle-hole symmetry breaking mechanism. It is not obvious that CAR is useful in the present case. In general, superconductors preserve particle-hole symmetry. I suggest that the Authors present a more insightful explanation on this important point. They quote Ref. 46. However, in that reference, quantum dots are considered in the contacts between a superconductor and normal leads and these are tunned in order to generate energy filters and break particle-hole symmetry. I do not see such an ingredient in the present case.
7. S3. No information is provided on the theoretical results presented in Fig. S3 (b). Which model was used?

Reviewer #3

(Remarks to the Author)
The paper explores electrical and thermoelectrical signals in hybrid quantum Hall / superconducting samples made in Cd3As2. The device geometry consists of QH region with a narrow SC finger familiar from the work of P. Kim’s group and others. The authors observe positive downstream resistance (Rd) which indicates that the normal electron processes (e.g.

cotunneling or some resistive process associated with vortices in the superconductor) dominate over the crossed Andreev reflections (CAR). In the main part of the paper, the authors apply a much larger current (200nA vs 1nA), which causes the second harmonic voltage to appear both downstream and upstream from the grounded superconductor. The origin of this signal has likely to be thermal: the large applied current locally increased the electron temperature, resulting the large thermoelectric signal proportional to the temperature increase. The appearance of the sizable thermoelectric signal roughly correlates with the Rd spots on the gate-B maps. However, the former signal changes sign with the magnetic field. The authors argue that the change of sign can reveal the presence of the CAR processes.

I am mostly convinced by the authors' data and presentation except for the crucial assumption in Figures 3 and S7. The authors propose that the normal contacts 1 and 2 are hot, while contact 3 is cold. I believe the opposite to be true. The Joule heating in the QH samples is divided evenly between the "hot spots" at the source and the drain. This power should heat the superconducting contact much more than the normal one, because the former cannot effectively dissipate heat. I believe this is why the signal disappears when the SC contact becomes normal. (Incidentally: the hot edge conducts negligible amount of energy and cannot substantially heat contact 2 even if contact 1 were cold, so the top edge would be cold regardless.)

If one assumes that the SC contact is hot, the origin of the positive/negative thermoelectric signal becomes more difficult to pinpoint. One can try to extend the authors' argument and think that the hot electrons on one side of the SC undergo two types of tunneling, normal and CAR, which have the opposite sign. The alternative explanation would be that the effective tunneling barrier (across the superconductor, or between the superconductor and the semiconductor) has a strong energy dependence, which results in a predominantly electron or hole signal. At this point, figuring out the microscopic origin of the signal is not trivial, and I do not have a clear suggestion of a measurement that could distinguish between the different mechanisms.

Other points:
1) The thermoelectric measurements in Figures 3b,d show that the thermoelectric signal is most negative at the highest magnetic fields and in the spin-polarized nu = 1 state. This suggests that CAR is strongest here when it is expected to be weakest.

2) In Figure S2, an increase in Rd is observed at DC voltage bias of about 20mV, which is attributed to suppressed superconductivity. However, 20mV is significantly larger than the estimated BCS gap of NbN (a BCS estimate based on a Tc of 6K yields 1 meV). The AC measurements as a 200nA excitation in the nu =1 state should result in a voltage drop of 5 mV (RMS?), also larger than the gap.

3) The colour scheme used in the maps saturates very easily, preventing estimates of the size of the measured signal. For example, see Rd at high field at nu =1 in Figure 1d. This also obscures the extent of the Landau levels which is important when comparing Rxx and Rd (Figures 1c,d for example).

4) Are there any measurements of the transparency of the superconducting interface? A low transparency may explain the positive Rd and would be informative in the interpretation of the thermoelectric signal.

Reviewer #4

(Remarks to the Author)
I co-reviewed this manuscript with one of the reviewers who provided the listed reports. This is part of the Nature Communications initiative to facilitate training in peer review and to provide appropriate recognition for Early Career Researchers who co-review manuscripts.

Version 1:

Reviewer comments:

Reviewer #1

(Remarks to the Author)
I thank the authors for their replies. I think, in particular, Table S1 is very useful. I note that for the 125 nm wide device "Sign-reversal" is still indicated as Y. Perhaps this could have an asterisk to further highlight the issues with finite resistance at 200 nA.

I was confused by the authors response to the other reviewer. They state that "a temperature difference is established by injecting hot electrons into the edge channels," implying the setup is equivalent to their previous work in Ref. 37. However, Ref. 37 used dedicated heaters and the authors' reply makes me confused as to whether this current device also has heaters. If the current device lacks heaters and relies on Joule heating from the bias current, then the thermal gradient in Figure 3a is likely reversed (as stated by the other reviewer). Standard dissipation in the quantum Hall effect occurs at the entry to the contacts; if this is the case here, the "hot" and "cold" edges are incorrectly labeled. The authors really need to clarify this since if the setup is different to Ref. 37 then the comparisons are misleading.

Reviewer #2

(Remarks to the Author)
Thermoelectric Detection of Crossed Andreev Reflections in Quantum Hall/Superconductor Hybrid Structures
Jiashu Wang, Sina Ahadi, Boliang Liu, Leon Balents, and Susanne Stemmer
I have read the new version of the manuscript and the supplementary information, as well as the responses to the Referees and the new reports.
I think that the Authors have successfully addressed most of the criticisms. In addition, I find this contribution very interesting, and the topic is very timely. However, I remain not fully convinced about the explanation of the role of the CAR in the particle-hole symmetry breaking mechanism. I agree with the authors that this mechanism is described by the joint density of states of the superconductors and the interfaces. Furthermore, following this reasoning I can conclude that the main ingredient is provided by the interface, rather than by the superconductor and the CAR.
I believe that another interpretation of the data is that the superconductor is heated because it hosts vortices, it acts as a hot terminal source, and the thermoelectric response is originated by the Landau levels acting as energy filters. Such a response has been measured in Corbino samples [see Phys.Rev. App. 14, 034019 (2020)] without superconductor. If that were the scenario, the role of the CAR is not essential. I think that the theoretical explanation provided in S3, as well as the results published in the earlier paper, by Huynh et al, agrees with this interpretation. Notice that thermoelectric effects induced by vortices heated by a current in a similar setup implemented in a graphene were reported in arXiv:2510.09798 and this scenario is akin the one suggested by Referees 3 and 4. Notice also that this should explain the response originated by the Landau levels, but not the response deep inside the plateaus. However, similar intriguing oscillating response within the plateaus were also reported in the Corbino system in 2020.
Before deciding about the recommendation of this article for publication, I would like the Authors to clarify this issue.
Finally, the Authors should check Fig. 3 (c). The meaningful measurement of the voltages for this configuration should be in the top edge instead of in the bottom.
A minor point: I found a typo in line 176: it should read ” formalism” instead of “formulism”.

Reviewer #3

(Remarks to the Author)
I have previously assumed that the schematics in Figure 3 should have the hot and the cold edges reversed, per my earlier report. The authors confirmed that their schematics is as intended. They refer to their group’s earlier paper [Huynh, Science Adv. (2025)]. That paper had dedicated heaters, and indeed the top edge emitted by the hot contact must have been hot. However, I understand that in the present paper, there are no dedicated heaters, and the sample is heated via Joule heating of the applied currents. Then the top edge in Figure 3a must be cold, and the dissipation should occur at the entry to the superconductor (also at the bottom left, entry point to the source contact). This is the conventional picture of the hot spots in the quantum Hall effect, see e.g. direct imaging by Kawano and Komiyama, Physica E (2000), the case corresponding to the low current regime: https://www.sciencedirect.com/science/article/pii/S1386947700000618
With that, I cannot recommend this paper for publication.

Reviewer #4

(Remarks to the Author)
I co-reviewed this manuscript with one of the reviewers who provided the listed reports. This is part of the Nature Communications initiative to facilitate training in peer review and to provide appropriate recognition for Early Career Researchers who co-review manuscripts.

Reviewer #5

(Remarks to the Author)
I co-reviewed this manuscript with one of the reviewers who provided the listed reports. This is part of the Nature Communications initiative to facilitate training in peer review and to provide appropriate recognition for Early Career Researchers who co-review manuscripts.

Version 2:

Reviewer comments:

Reviewer #1

(Remarks to the Author)
The explanation of the hot electron injection largely resolves the confusion regarding how the temperature gradient actually forms. The authors have adequately addressed my concerns.

Reviewer #2

(Remarks to the Author)

I have read the new version of the manuscript as well as the responses by the Authors. I believe that the Authors addressed appropriately all the criticism and that the present version of the manuscript satisfies the criteria for being published in Nature Communication.

Reviewer #4

(Remarks to the Author)
I co-reviewed this manuscript with one of the reviewers who provided the listed reports. This is part of the Nature Communications initiative to facilitate training in peer review and to provide appropriate recognition for Early Career Researchers who co-review manuscripts.

Open Access This Peer Review File is licensed under a Creative Commons Attribution 4.0 International License, which permits use, sharing, adaptation, distribution and reproduction in any medium or format, as long as you give appropriate credit to the original author(s) and the source, provide a link to the Creative Commons license, and indicate if changes were made.
In cases where reviewers are anonymous, credit should be given to 'Anonymous Referee' and the source.
The images or other third party material in this Peer Review File are included in the article’s Creative Commons license, unless indicated otherwise in a credit line to the material. If material is not included in the article’s Creative Commons license and your intended use is not permitted by statutory regulation or exceeds the permitted use, you will need to obtain permission directly from the copyright holder.
To view a copy of this license, visit https://creativecommons.org/licenses/by/4.0/

NCOMMS-25-73772
Response to referee's comments and summary of changes made

Referee 1 comments	Authors' response/changes made
Wang et al. report on the detection of Crossed Andreev Reflections (CAR) in a Cd3As2/NbN hybrid structure using thermoelectric measurement in the quantum Hall regime. First they note that the downstream resistance (Rd) is either approximately zero or positive depending on device geometry e.g. width. They attribute this to an approximate cancellation of CAR with electron cotunnelling (ECT). Next, they observe oscillations in what they call the thermoelectric voltage $V_d^{2\omega}$, with sign changes in the lower Landu levels interpreted as a signature of dominant CAR. While the topic is timely and the conclusions about thermal transport signatures of CAR would be very interesting/useful for the field if convincingly demonstrated, I have significant concerns about the reproducibility of the main effect and how this impacts the interpretation. Furthermore, clarification is needed regarding the origin of the non-linear signal. Given these core issues, at this stage, I cannot recommend publication in Nature Communications.	We thank the reviewer for the careful reading and useful suggestions for improvement. We respond to each question in detail below.
1. My primary concern is the reproducibility of the sign-alternating thermoelectric voltage, which is the central claim of the manuscript.<br>• The authors state that the effect is observed in "many different devices" with NbN strips narrower than 200 nm. However, this claim appears to be contradicted by the supplementary information. Figure S6 shows data from three additional devices with identical 175 nm widths, yet only one of them clearly exhibits the sign-reversing oscillations in $V_d^{2\omega}$. This inconsistency makes me quite confused about the robustness of the phenomenon. What does "many devices" mean here? How often is this effect not seen? Some idea is required to put the claim into context.	We thank the reviewer for the suggestion to discuss the reproducibility. Indeed, the non-zero R_d and $V_d^{2\omega}$ and the oscillations are not always present in all devices, particularly for devices with wider fingers, such as the 175 nm devices, shown in Fig. S6. The width dependence was already discussed in the original manuscript. Figure S6 was shown to illustrate this: we showed three representative cases (strong, weak and no oscillations). In addition, Fig. S6 served to show the link between oscillations in R_d and $V_d^{2\omega}$. In the revised manuscript, we summarize the observations for all investigated devices for each width. We have also added a list in a Table in the Supplement.<br>As already noted in the original manuscript, the fact that the oscillations are more prominent and more frequently observed in thinner fingers is consistent with tunneling/CAR origin. Furthermore, for reasons now discussed more extensively in the revised manuscript (see also our responses to the other reviewers) it is likely that the tunneling/CAR is quite sensitive to the nature of the interface, which may also vary between devices.

• Furthermore, I cannot find in either in the supplemental material or main text the thermoelectric data for other device widths shown in the main text (e.g., the 125 nm device, which shows the strongest Rd oscillations, and the wider devices that do not show this). The authors should provide a table of all measured devices. This table should list device parameters (e.g., NbN width) and clearly state whether the key effects (oscillations in Rd and sign reversal in $V_d^{2\omega}$) were observed, pointing to the relevant figure for each device.	We thank the reviewer for the suggestion and we have added a list to the supplement of all devices and a summary of the observations (Table S1)<br><br>We did not present the $V_d^{2\omega}$ for the 125 nm device, because at excitation currents of 200 nA (used for the $V_d^{2\omega}$ measurements) and high magnetic fields, this device showed a finite resistance and was only partially superconducting. We mention this in the supplement.
It would also be useful to add device name (or at least width) to the figures of $V_d^{2\omega}$.	We thank the reviewer for this suggestion. We have added the width to Fig. 3. We also add the device name to the $V_d^{2\omega}$ map in supplementary figures S6 and S7.
2. Data Noise: The differential resistance data shown in Fig. S2 appears to have a very low signal-to-noise ratio. Could the authors comment on the large fluctuations in these bias-dependent measurements? A clear signal is essential for interpreting the non-linear response.	In these bias measurements, we are applying a large DC current while reading the ac response of a small ac current (1 nA). The noise level is around 150 nV. If we divide by 1 nA it leads to 150 Ohm of noise, much larger than in all other measurements. The main feature, which is above the noise level, remained unchanged during multiple sweeps. We thus think the result is robust despite the noise. The main experiments, namely R_d and $V_d^{2\omega}$ measurements, did not have a DC bias and have a good noise level (below 50 nV). The magnitude of main signal in R_d (hundreds of Ohms in oscillations) and $V_d^{2\omega}$(a few microvolts) is much larger than the noise level.
3. Origin of the 2ω signal: The authors should exclude other effects known to produce 2ω, such as magnetochiral anisotropy (MChA), which are plausible in a chiral material like Cd3As2 and can produce a 2ω signal. In their previous work (Huynh et al., Sci. Adv. 2025), the signal was explicitly tested for B-field symmetry. Can a similar symmetrisation/antisymmetrisation be performed here? I appreciate there is already a difference in Landau level chirality for ±B. However, given the signal to noise ratio (see above), I worry that this effect is not actually related to thermoelectric currents, but something else like MChA or even a very trivial effect like a non-Ohmic contact (the exclusion of which could also do with a bit of discussion).	We did test the symmetry in magnetic field (Fig. 3 and Fig. S7). In case of MChA, the effect is odd with magnetic field. In our case, the sign of $V^{2\omega}$ does not change with field direction (Figs. 3 and S7). The nonlinear Hall effect is also odd with field. We also note that the conditions for MChA (inversion symmetry breaking) are not fulfilled. Cd_3As_2 is not a chiral material and it is centrosymmetric. Moreover, the samples are clearly in the quantum Hall regime. We have added a sentence along these lines to the manuscript.<br><br>In addition to the Huynh paper, we have extensively studied thermoelectric signals in Hall bars with non-superconducting contacts as part of a different study investigating ballistic heat transport, which is currently under review elsewhere. We would be happy to share this manuscript with the reviewer.

	For a non-Ohmic contact, we would observe a finite resistance in the first-order measurement. Such resistance is usually independent, or monotonically dependent, on the magnetic field and does not scale with filling factor (as seen in Fig. S1d). This was already discussed in the original manuscript. The signal-to-noise ratio has been discussed in the response to the previous question. For R_d and $V_d^{2\omega}$ measurements, the magnitude of the main feature is much larger than the noise level.
Given these issues, especially around the reproducibility, I would not feel comfortable recommending publication in Nature Communications.	We hope that the extra explanations and additional data have addressed the reviewer's concerns.

**Reviewer 2 comments**	**Author's response/Changes made**
The authors present experimental results on the electric and thermoelectric transport in edge quantum Hall edge modes of cadmium arsenide with a thin superconducting NbN strip. They find downstream resistance oscillation as a function of the magnetic field, indicative of the competition between elastic cotunneling (ECT) and cross Andreev reflection (CAR). They measure the thermoelectric response and identify signatures of hole-like carriers.<br>The topic is very interesting and timely. The experimental results are very carefully presented and analyzed. I believe that the present contribution has the potential for being published in Nature Communication. However, before recommending its publication I suggest the Authors to revise the manuscript and carefully address the following points:	We thank the reviewer for the careful reading of the manuscript, the positive comments, and the very useful suggestions for clarification and improvement.
1. Fig. 1 a: Please check the edge state injected from V_d and propagating to the left. I think it should be drawn as a solid blue line.	Thank you and we have corrected it.
2. I suggest indicating the temperature in the Figs, although it is reported in the section "Methods".	Thank you. We have added the temperature information to Figs.1 and 3.
3. Before Eq. 1, the Authors state that "in the absence of CAR and/or ECT R_d equals the longitudinal resistance R_{xx}". Is this also true in the presence of chiral Andreev states (CAES) circulating along all the length of width of the finger? If the dimensions of the finger rule out the existence of such states, the Authors should mention and discuss this.	The reviewer is correct, CAES can also produce a non-zero R_d. As already discussed in the original manuscript, if CAES plays a role, we would not expect a small change of width (25 nm) to cause large changes in R_d given the total length (>10 μm ~ circumference). In addition, when the length of the SC is significantly longer than the skipping orbit, holes and electrons are balanced (resulting in

	zero R_d) due to multiple Andreev reflections. This has been discussed in refs. [16-18]. We now mention this point in the revised manuscript. Given both points above, we do not think the results are due to CAES.
4. In Page 7 it is mentioned that at high magnetic fields, spin polarization of the edge states makes CAR difficult. This is true. However, they mentioned in the introduction that these films have a large spin-orbit and this could change their conclusion [see PRB 83, 195441 (2011), Ref. 28 of the present manuscript and PRB 109, 024504 (2012)].	We appreciate the reviewer for raising this question. We believe that at large magnetic fields the Zeeman effect overwhelms SOC, and have added a remark along these lines to the revised manuscript. We would like to emphasize, however, that we do not have any measurements of these quantities, making these arguments somewhat speculative.
5. Fig.3. The features observed within the $\nu=2$ and higher filling factors look quite different in panels (b) and (d). Do the Authors have an explanation for this behavior? The agreement seems to be better for the results presented in Fig. S7.	The device shown in Fig. 3 underwent many high-current experiments in the course of this study, such as the bias measurements with current exceeding 1 μA in supplement S4. The purpose was to conduct as many of the measurements on the same device, but the large current experiments inevitably led to some deterioration. Noting these changes, we conducted studies on another device that had undergone fewer measurement cycles, shown in Fig. S7. In this case, both field directions look similar. We added a sentence to the revised manuscript.
6. In order to have a thermoelectric response, it is necessary to have a particle-hole symmetry breaking mechanism. It is not obvious that CAR is useful in the present case. In general, superconductors preserve particle-hole symmetry. I suggest that the Authors present a more insightful explanation on this important point. They quote Ref. 46. However, in that reference, quantum dots are considered in the contacts between a superconductor and normal leads and these are tunned in order to generate energy filters and break particle-hole symmetry. I do not see such an ingredient in the present case.	We agree with the reviewer about the thermoelectric response of a particle-hole symmetric superconductor. However, the tunneling probability should also involve the density of states outside of the superconductor to some degree. In a naïve picture, the transmission matrix is product of the superconducting DOS and the DOS at the interface, which is not necessarily particle-hole symmetric. We thank the referee for making this important point and have added additional discussion to the revised manuscript.
7. S3. No information is provided on the theoretical results presented in Fig. S3 (b). Which model was used?	The thermoelectric effects in the quantum Hall regime and the Mott relationship were obtained by Jonson and Girvin [*Phys. Rev. B* **29**, 1939-1946 (1984).] We have expanded the discussion in the Supplement.

**Reviewer 3 comments**	**Author's response/Changes made**
The paper explores electrical and thermoelectrical signals in hybrid quantum Hall / superconducting samples made in Cd3As2. The device geometry consists of QH region with a narrow SC finger	Thank you for carefully reading of our manuscript, the positive comments and the very useful suggestions for improvement.

familiar from the work of P. Kim's group and others. The authors observe positive downstream resistance (Rd) which indicates that the normal electron processes (e.g. cotunneling or some resistive process associated with vortices in the superconductor) dominate over the crossed Andreev reflections (CAR). In the main part of the paper, the authors apply a much larger current (200nA vs 1nA), which causes the second harmonic voltage to appear both downstream and upstream from the grounded superconductor. The origin of this signal has likely to be thermal: the large applied current locally increased the electron temperature, resulting the large thermoelectric signal proportional to the temperature increase. The appearance of the sizable thermoelectric signal roughly correlates with the Rd spots on the gate-B maps. However, the former signal changes sign with the magnetic field. The authors argue that the change of sign can reveal the presence of the CAR processes.	
I am mostly convinced by the authors' data and presentation except for the crucial assumption in Figures 3 and S7. The authors propose that the normal contacts 1 and 2 are hot, while contact 3 is cold. I believe the opposite to be true. The Joule heating in the QH samples is divided evenly between the "hot spots" at the source and the drain. This power should heat the superconducting contact much more than the normal one, because the former cannot effectively dissipate heat. I believe this is why the signal disappears when the SC contact becomes normal. (Incidentally: the hot edge conducts negligible amount of energy and cannot substantially heat contact 2 even if contact 1 were cold, so the top edge would be cold regardless.)	We apologize for the misleading wording and Figure color scheme in the Supplementary. To clarify, all voltage probes are at the bath temperature (= cold). There is no heat loss along the edge of the Hall bar until the carriers equilibrate at the cold drain. When we wrote "hot contacts" and "cold contacts" in the Supplementary, this was meant to refer to the temperature of the edge states at those contacts and for the purpose of the LB model. Referring to Fig. 3a or S7, the edge states on the upper edge of the Hall bar are hot because they are Joule heated at the interface with the source contact. They equilibrate to the bath temperature at the drain, which is the superconducting contact, which is cold. The direction of the temperature gradient is reflected in the signs of $V_x^{2\omega}$ and $V_y^{2\omega}$ as shown in W. Huynh, Science Adv. 11, eady9006 (2025). In another manuscript (submitted elsewhere, but happy to share with the referee), where all the contacts are normal, we have also shown that the edge states remain hot until they reach the drain, where they equilibrate. The bottom edge is in equilibrium with the cold drain (= the superconducting contact). Thus, regardless of whether the drain is normal or superconducting there is a thermal gradient across it. We have modified the wording in the Supplement and its figures to clarify that the

	voltage probes are cold and the thermal gradient is established via the edge states.<br>We have considered the possibility that there might be some heating near the superconductor interface due to the need to dissipate the heat there for equilibration. This was addressed in the second half of the extended LB model in the Supplement. We have clarified the wording and added the calculation (in the original supplement only the result was shown).
If one assumes that the SC contact is hot, the origin of the positive/negative thermoelectric signal becomes more difficult to pinpoint. One can try to extend the authors' argument and think that the hot electrons on one side of the SC undergo two types of tunneling, normal and CAR, which have the opposite sign. The alternative explanation would be that the effective tunneling barrier (across the superconductor, or between the superconductor and the semiconductor) has a strong energy dependence, which results in a predominantly electron or hole signal. At this point, figuring out the microscopic origin of the signal is not trivial, and I do not have a clear suggestion of a measurement that could distinguish between the different mechanisms.	To clarify, the SC contact is cold, see above. The argument in the original manuscript was exactly what the reviewer describes.
Other points:<br>1) The thermoelectric measurements in Figures 3b,d show that the thermoelectric signal is most negative at the highest magnetic fields and in the spin-polarized nu = 1 state. This suggests that CAR is strongest here when it is expected to be weakest.	As discussed in the manuscript, a negative signal is attributed to CAR not ECT. We think the confusion comes from a missing minus sign in Eq. 3, which we have corrected. We apologize for this. A negative thermoelectric signal indicates a positive $\partial(P_e - P_h)/\partial E$, which indicates ECT dominates at high magnetic field, consistent with R_d.
2) In Figure S2, an increase in Rd is observed at DC voltage bias of about 20mV, which is attributed to suppressed superconductivity. However, 20mV is significantly larger than the estimated BCS gap of NbN (a BCS estimate based on a Tc of 6K yields 1 meV). The AC measurements as a 200nA excitation in the nu =1 state should result in a voltage drop of 5 mV (RMS?), also larger than the gap.	The data in Fig. S2 suggests that the observed feature is associated with a superconducting transition: it is suppressed with increasing magnetic field, and the critical voltage is consistently larger for $\nu = 1$ than for $\nu = 2$. That said, we agree with the referee that the applied voltage in both cases is larger than the superconducting gap of NbN.<br>In the revised manuscript and supplementary, we have added a possible explanation. In particular, given the presence of an accumulation layer at the NbN interface, it is possible that some voltage drops occurs before the electrons reach the superconducting interface, if this accumulation layer is not fully proximitized. Consequently, the effective interfacial potential depends on ν' (filling

	factor near the interface) rather than on the bulk filling factor v. Since $v' > v$, this effect is not directly reflected in the total resistance. This scenario may also explain why the CAR signal is significantly weaker in our devices. We thank the referee for making this point.
3) The colour scheme used in the maps saturates very easily, preventing estimates of the size of the measured signal. For example, see Rd at high field at nu =1 in Figure 1d. This also obscures the extent of the Landau levels which is important when comparing Rxx and Rd (Figures 1c,d for example).	We thank the referee for noting this and have adjusted the scale of Figs. 1d and Fig. S5. Other figures do not seem to have the scale issue.
4) Are there any measurements of the transparency of the superconducting interface? A low transparency may explain the positive Rd and would be informative in the interpretation of the thermoelectric signal.	We are not aware of any approach for superconducting/QH interfaces. We made NbN Josephson junctions on Cd_3As_2 films that were not in the QH state and were able to estimate transparency based on the excess current. From these junctions, we got a transparency of $\tau = 0.27$. We have added this to the Supplementary.

Response Letter to Second Round of Reviews
Manuscript NCOMMS-25-73772A

First of all, we would like to thank all referees for the second careful reading and their comments. All three reports have questions with regards to the direction of the temperature gradient and how it is established (Reviewers #1 and #3) and, related, if the superconducting contact is heated, because of vortices (Reviewer #2). In addition, reviewer #2 suggests that the device could function like a Corbino disk. We address the first point first and then comment on the Corbino disk.

1. Temperature gradient and hot-electron Hall bar device

This section responds to the following comments:

Reviewer #1: I was confused by the authors response to the other reviewer. They state that "a temperature difference is established by injecting hot electrons into the edge channels," implying the setup is equivalent to their previous work in Ref. 37. However, Ref. 37 used dedicated heaters and the authors' reply makes me confused as to whether this current device also has heaters. If the current device lacks heaters and relies on Joule heating from the bias current, then the thermal gradient in Figure 3a is likely reversed (as stated by the other reviewer). Standard dissipation in the quantum Hall effect occurs at the entry to the contacts; if this is the case here, the "hot" and "cold" edges are incorrectly labeled. The authors really need to clarify this since if the setup is different to Ref. 37 then the comparisons are misleading.

Reviewer #3: I have previously assumed that the schematics in Figure 3 should have the hot and the cold edges reversed, per my earlier report. The authors confirmed that their schematics is as intended. They refer to their group's earlier paper [Huynh, Science Adv. (2025)]. That paper had dedicated heaters, and indeed the top edge emitted by the hot contact must have been hot. However, I understand that in the present paper, there are no dedicated heaters, and the sample is heated via Joule heating of the applied currents. Then the top edge in Figure 3a must be cold, and the dissipation should occur at the entry to the superconductor (also at the bottom left, entry point to the source contact). This is the conventional picture of the hot spots in the quantum Hall effect, see e.g. direct imaging by Kawano and Komiyama, Physica E (2000), the case corresponding to the low current regime: https://www.sciencedirect.com/science/article/pii/S1386947700000618

Hot electron injection *from the source contact* has been used for heating edge states in the quantum Hall literature, see, e.g., PRL 102, 086803 (2009), PRL 110, 226801 (2013), Sci. Adv. 5, eaaw5798 (2019). Because the method is established, we cited the literature, instead of elaborating in detail.

Here, we summarize the principles of the hot electron injection method to heat the edge states. In the aforementioned studies, as in the present manuscript, the edge states are heated at the *source contact* via Joule heating. A large current I injected at the source heats the contact with power:

$$P = I^2 R = I^2 h/\nu_b e^2 \qquad \text{(R1)}$$

where R is the interface resistance, determined by the bulk filling factor ν_b. The edge mode leaves the source with chemical potential $\mu = I\frac{h}{\nu_b e^2}$ and electron-electron scattering quickly

thermalizes the modes into a hot Fermi-Dirac distribution with electron temperature T_S. Thus, as indicated in Fig. 3a, the *high potential edge is the hot edge.* Reversing the direction of the field (Fig. 3c) reverses which edge is hot, due to the chirality of the edge states.

We have added the above discussion to the revised supplementary (beginning of Section S3).

Many experiments in the literature have confirmed this. Because the thermopower is zero in the quantum Hall plateaus, the two PRLs referenced above added additional "sensor gates" along the edges. If the sensor gate is tuned into a Landau level, there is a thermal gradient across it and a thermoelectric voltage is measured. We have done similar experiments, which are the subject of a submitted manuscript, which is, unfortunately, still under review, but we show some of the results here.

Figure R1 shows results from a Hall bar made on a similar film as the one discussed in the manuscript. Contacts 5 and 6 are operated as source or drain, respectively, with the field oriented as in Fig. 3a. In the manuscript, the *superconductor is drain contact*. In Fig. R1, the thermoelectric voltage is measured between contacts 1 and 7, while the sensor gate G1 is tuned through the Landau levels and Hall plateaus. The thermoelectric response is zero in the Hall plateaus. With contact 5 as the source contact, the upper edge is the high potential edge; thus G1 resides on the hot edge and a thermoelectric response is measured (a thermal gradient develops across G1 due to heat dissipation when the Fermi level is in a Landau level). When contact 6 is the source, G1 resides on the cold edge, no thermal gradient is established, and the thermoelectric signal is zero for all G1 voltages. The configuration shown in Fig. 3a in the manuscript corresponds to the top schematic shown in Fig. R1 (with contact 6 being the superconductor) – i.e., the edge channels that are upstream from the superconductor are hot.

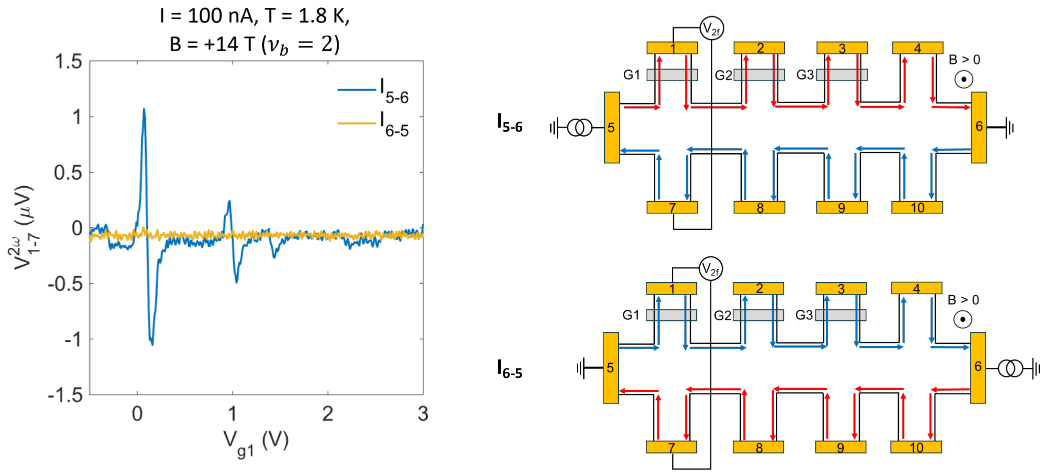

Figure R1

The thermoelectric effects of the Hall bar device (Fig. S3) are consistent with these results, theoretical predictions, and the literature (see also discussion below).

Further tests confirming the heating at the source contact are also possible. For example, Fig. R2 shows the thermoelectric voltage as a function of power P. The thermoelectric response is proportional to the temperature gradient and, in case of Joule heating, the power. Here, the power is calculated as $P = I_{56}^2 R_{56}$, where R_{56} was modulated by changing the bulk filling factor (see equation R1 above). Increasing the current causes more heating of the *source contact*, which raises T_s along with μ. The signal scales quadratically with current as expected for Joule heating ($P = I^2 R$). This suggests that heating primarily occurs at the point of current injection. We note that all of this in no way contradicts the hot spot picture of the quantum Hall regime.

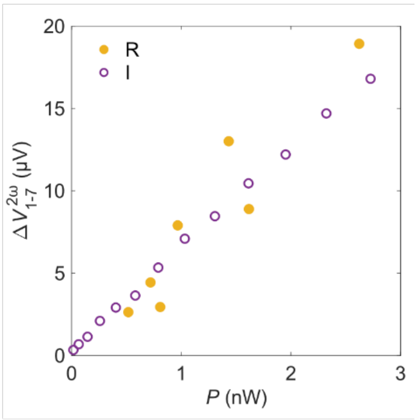

Figure R2

To summarize, heating occurs primarily at the source contact, with the power dissipated at the source contact being proportional to the interface resistance, which, on a Hall plateau, is controlled by the number of edge channels.

Referee #2 suggests that the superconducting contact may be heated because of the presence of vortices. Note again that the superconducting contact is the drain. We can consider a normal drain contact, discussed above, as being an extreme case of a superconductor with lots of vortices. In the experiments discussed above the possibility of heating by the drain contact is directly tested via the sensor gates downstream from the drain (bottom configuration in Fig. R1): there is no indication of heating (thermoelectric effects) in the channels downstream from the drain. Finally, we note that the main features in the downstream resistance, measured with very small currents that do not cause heating, align with the thermoelectric features, and do not change measurably even under larger currents, as discussed in the manuscript.

2. <u>Thermoelectric Properties of a Corbino Disk</u>

Referee #2 suggests the following scenario: "*I believe that another interpretation of the data is that the superconductor is heated because it hosts vortices, it acts as a hot terminal source, and the thermoelectric response is originated by the Landau levels acting as energy filters. Such a response has been measured in Corbino samples [see Phys.Rev. App. 14, 034019 (2020)] without superconductor.*"

We have already discussed the direction of the temperature gradient above. To respond to the question about Landau levels and Corbino disks, we now discuss in detail the thermoelectric properties of Hall bars and Corbino disks.

At the outset we note that the device studied here is a Hall bar. The measurements of interest for detecting CAR and ECT are when the Hall bar is tuned into a quantum Hall plateau, i.e., the Fermi level is between Landau levels. In a Hall bar, the *longitudinal resistance* (R_{xx}) is zero in the quantum Hall plateaus, as clearly seen (green regions) in Fig. 1c. In contrast, R_{xx} peaks when the Fermi level is in the Landau levels. The thermoelectric response of Hall bar samples is well established, see Phys. Rev. B 29, 1939 (1984) for the theory, which has been confirmed in many experiments. In particular, both longitudinal and transverse components of the thermoelectric tensor are **zero in the Hall plateaus** and non-zero in the Landau levels (Fig. R3).

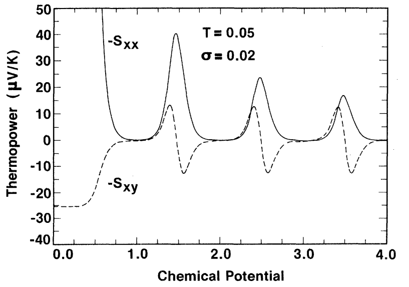

FIG. 2. Diagonal and off-diagonal components of the thermopower tensor vs chemical potential in units where $\hbar\omega_c = 1$, $k_B T = 0.05$, and $\hbar\sigma = 0.02$. If $B = 12$ T this corresponds to $T = 4.3$ K and a Landau-level width ($4\hbar\sigma$) of 0.6 meV (6.9 K).

Figure R3: Thermoelectric response of a Hall bar: from Phys. Rev. B 29, 1939 (1984) for comparison with Fig. S3.

As shown in Supplementary Figure S3, the Hall bar studied in our manuscript behaves exactly as expected from Fig. R3: the thermoelectric response is zero in the Hall plateaus and shows oscillating behavior in the Landau levels (note that our thermal gradient, established by the edge states, is across the width of the Hall bar, while $V_x^{2\omega}$ is measured along the edges, normal to the thermal gradient, making $V_x^{2\omega}$ a Nernst signal, or S_{xy} in the notation of Fig. R3, see discussion in the Supplement).

Thus, indeed, the thermoelectric response is non-zero in the Landau levels. However, the *downstream responses are measured while the Hall bar was in a Hall plateau. Moreover, oscillations in the Landau levels behave very differently from our downstream signal:* the signal oscillates *as the gate voltage is swept across the Landau level*, as seen in Fig. R3 and S3. In contrast, the downstream thermoelectric response measured when the sample is in a Hall plateau does not sharply oscillate as the Fermi level is swept across the plateau. In summary, our device functions as expected for a Hall bar device, both in its electrical and its thermoelectric behavior.

We have added a few sentences along these lines to the revised manuscript.

In a **Corbino disk**, the situation is different. The radial resistance, R_{rr}, peaks at the center of the Hall plateaus. As discussed above, our Hall bar shows zero longitudinal resistance in the Hall plateaus and behaves like a Hall bar, not like a Corbino disk. The difference is caused by the fact that in the Corbino disk, G_{rr} is minimized in the Hall plateaus, and is inversely related to R_{rr}. Another way to say this is that the transport across the Corbino disk is edge-free, i.e, the edge states do not contribute. This (via the Mott formula), gives rise to the peculiar behavior of a Corbino disk's thermoelectric behavior, shown in Fig. R4: there are sawtooth oscillations of the

Corbino thermopower near the center of the Hall plateaus, in addition to oscillations in the Landau levels.

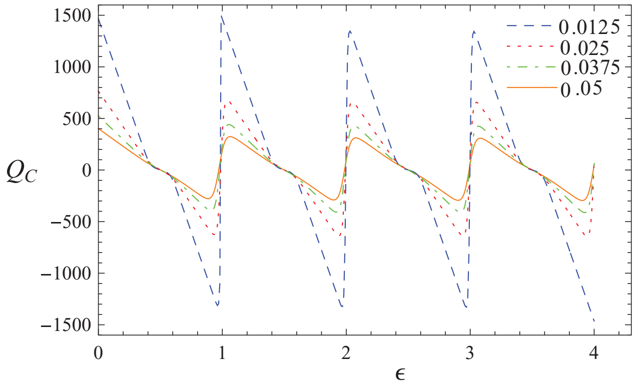

FIG. 5. (Color online) Corbino thermopower calculated for fixed disorder $\hbar\sigma = 0.05$ in units of $\hbar\omega_c$ at different temperatures $k_\beta T =$ 0.0125, 0.025, 0.375, and 0.05. The thermopower Q_C is measured in μ V/K and the chemical potential ϵ is in units of $\hbar\omega_c$.

Figure R4: Thermoelectric response of a Corbino disk: from Phys. Rev. B **85**, 195107 (2012). The numbers on the x-axis indicate the filling factor. Moving along the x-axis (ε) corresponds to a gate voltage sweep.

The Corbino disk behavior shown in Fig. R4 bears no resemblance to our data: in our data $V_x^{2\omega}$ is zero across the entire Hall plateau, unlike $V_r^{2\omega}$ of a Corbino disk. When the Fermi level is within a Hall plateau, we observe a non-zero downstream thermoelectric response, as discussed in the manuscript; however, this response *does not* show the sharp oscillations of a Corbino disk when the Fermi level is swept across the plateau. We have data on Corbino disks that look like expected from Fig. R4, and would be happy to share.

3. Comparison with arXiv:2510.09798

Referee #2 suggests that "*notice that thermoelectric effects induced by vortices heated by a current in a similar setup implemented in a graphene were reported in arXiv:2510.09798*".

The data in *arXiv:2510.09798*, while very interesting, bears no resemblance to our data. In *arXiv:2510.09798* fairly high currents are used, causing heating effects, while we measure a downstream voltage also with very small currents, when there is no heating. We also confirmed that the features in the downstream electric voltage were independent of the currents used (see Fig. S5), indicating that heating plays no role in the features of R_d. The features observed in *arXiv:2510.09798* are also very different, with small changes in the gate voltage causing rapid fluctuations. We do not observe this in our thermoelectric measurements. We see a single oscillation at the Landau level, well described by expectations from theory, as discussed above. Unlike *arXiv:2510.09798* we do not see any rapid fluctuations with gate voltage in the plateaus. Unlike *arXiv:2510.09798*, our observations are stable in magnetic field over many, many measurements. While we do not rule out heating at the top edge of our superconductor, this does not change the picture, as discussed in the manuscript (Eq. 3 and S6).

We have added a sentence mentioning that the downstream electric signal is not thermoelectric in origin.

4. <u>Additional responses</u>

Reviewer #1: I note that for the 125 nm wide device "Sign-reversal" is still indicated as Y. Perhaps this could have an asterisk to further highlight the issues with finite resistance at 200 nA.

We thank the reviewer for the suggestion. We have added asterisks to table S1.

Reviewer #2: Finally, the Authors should check Fig. 3 (c). The meaningful measurement of the voltages for this configuration should be in the top edge instead of in the bottom.

The goal of Figs. 3(a, c) is to verify our theoretical picture that the upstream voltage is equal to the downstream voltage. By reversing the field direction, the bottom edge now becomes the "upstream edge." The comparison is only meaningful if measure on the same contacts. Please also see the discussion above.

Reviewer #2: I found a typo in line 176: it should read " formalism" instead of "formulism".

We thank the reviewer for noticing. This has been corrected.

Review of Wang, Ahadi, Stemmer

Wang et al. report on the detection of Crossed Andreev Reflections (CAR) in a Cd3As2/NbN hybrid structure using thermoelectric measurement in the quantum Hall regime. First they note that the downstream resistance (R_d) is either approximately zero or positive depending on device geometry e.g. width. They attribute this to an approximate cancellation of CAR with electron cotunnelling (ECT). Next, they observe oscillations in what they call the thermoelectric voltage ($V_d^{2\omega}$), with sign changes in the lower Landu levels interpreted as a signature of dominant CAR.

While the topic is timely and the conclusions about thermal transport signatures of CAR would be very interesting/useful for the field if convincingly demonstrated, I have significant concerns about the reproducibility of the main effect and how this impacts the interpretation. Furthermore, clarification is needed regarding the origin of the non-linear signal. Given these core issues, at this stage, I cannot recommend publication in Nature Communications.

1. My primary concern is the reproducibility of the sign-alternating thermoelectric voltage, which is the central claim of the manuscript.

- The authors state that the effect is observed in "many different devices" with NbN strips narrower than 200 nm. However, this claim appears to be contradicted by the supplementary information. Figure S6 shows data from three additional devices with identical 175 nm widths, yet only one of them clearly exhibits the sign-reversing oscillations in $V_d^{2\omega}$. This inconsistency makes me quite confused about the robustness of the phenomenon. What does "many devices" mean here? How often is this effect not seen? Some idea is required to put the claim into context.
- Furthermore, I cannot find in either in the supplemental material or main text the thermoelectric data for other device widths shown in the main text (e.g., the 125 nm device, which shows the strongest R_d oscillations, and the wider devices that do not show this).

The authors should provide a table of all measured devices. This table should list device parameters (e.g., NbN width) and clearly state whether the key effects (oscillations in R_d and sign reversal in $V_d^{2\omega}$) were observed, pointing to the relevant figure for each device. It would also be useful to add device name (or at least width) to the figures of $V_d^{2\omega}$.

2. **Data Noise:** The differential resistance data shown in Fig. S2 appears to have a very low signal-to-noise ratio. Could the authors comment on the large fluctuations in these bias-dependent measurements? A clear signal is essential for interpreting the non-linear response.

3. **Origin of the 2ω signal:** The authors should exclude other effects known to produce 2ω, such as magnetochiral anisotropy (MChA), which are plausible in a chiral material like Cd3As2 and can produce a 2ω signal. In their previous work (Huynh et al., *Sci. Adv.* 2025), the signal was explicitly tested for B-field symmetry. Can a similar symmetrisation/antisymmetrisation be performed here? I appreciate there is already a difference in Landau level chirality for $\pm B$. However, given the signal to noise ratio (see above), I worry that this effect is not actually related to thermoelectric currents, but something else like MChA or even a very trivial effect like a non-Ohmic contact (the exclusion of which could also do with a bit of discussion).

Given these issues, especially around the reproducibility, I would not feel comfortable recommending publication in Nature Communications.
